# Transcription factor 4 maintains endothelial cell identity by inhibiting endothelial to mesenchymal transition

**DOI:** 10.1093/nar/gkaf931

**Published:** 2025-10-29

**Authors:** Gaopeng Xian, Rongbin Zheng, Jie Lv, Sen Zhu, Min Chen, Xinlei Gao, Shenli Yuan, Zhen Bouman Chen, Keith Youker, John P Cooke, Kaifu Chen, Lili Zhang

**Affiliations:** Basic and Translational Research Division, Department of Cardiology, Boston Children's Hospital, Boston, MA 02115, United States; Department of Pediatrics, Harvard Medical School, Boston, MA 02115, United States; Basic and Translational Research Division, Department of Cardiology, Boston Children's Hospital, Boston, MA 02115, United States; Department of Pediatrics, Harvard Medical School, Boston, MA 02115, United States; Center for Bioinformatics and Computational Biology, Department of Cardiovascular Sciences, Houston Methodist Research Institute, Houston, TX 77030, United States; Center for Bioinformatics and Computational Biology, Department of Cardiovascular Sciences, Houston Methodist Research Institute, Houston, TX 77030, United States; Basic and Translational Research Division, Department of Cardiology, Boston Children's Hospital, Boston, MA 02115, United States; Department of Pediatrics, Harvard Medical School, Boston, MA 02115, United States; Basic and Translational Research Division, Department of Cardiology, Boston Children's Hospital, Boston, MA 02115, United States; Department of Pediatrics, Harvard Medical School, Boston, MA 02115, United States; Basic and Translational Research Division, Department of Cardiology, Boston Children's Hospital, Boston, MA 02115, United States; Department of Pediatrics, Harvard Medical School, Boston, MA 02115, United States; Department of Diabetes Complications and Metabolism, City of Hope, Duarte, CA 91010, United States; Center for Cardiovascular Regeneration, Department of Cardiovascular Sciences, Houston Methodist Research Institute, Houston, TX 77030, United States; Center for Cardiovascular Regeneration, Department of Cardiovascular Sciences, Houston Methodist Research Institute, Houston, TX 77030, United States; Basic and Translational Research Division, Department of Cardiology, Boston Children's Hospital, Boston, MA 02115, United States; Department of Pediatrics, Harvard Medical School, Boston, MA 02115, United States; Center for Bioinformatics and Computational Biology, Department of Cardiovascular Sciences, Houston Methodist Research Institute, Houston, TX 77030, United States; Center for Cardiovascular Regeneration, Department of Cardiovascular Sciences, Houston Methodist Research Institute, Houston, TX 77030, United States; Basic and Translational Research Division, Department of Cardiology, Boston Children's Hospital, Boston, MA 02115, United States; Department of Pediatrics, Harvard Medical School, Boston, MA 02115, United States; Center for Cardiovascular Regeneration, Department of Cardiovascular Sciences, Houston Methodist Research Institute, Houston, TX 77030, United States

## Abstract

Endothelial to mesenchymal transition (EndoMT) is essential for embryonic heart development and contributes to many pathological processes. It is unclear how the balance between endothelial cell (EC) identity and EndoMT mediators is regulated to drive this transition. This study identifies transcription factor 4 (*TCF4*; also known as *ITF2*) as a critical EC identity gene. TCF4 knockdown impairs EC phenotype and function, and induces a transition towards a mesenchymal-like state. This discovery suggests that TCF4 safeguards EC identity against EndoMT. Mechanistically, TCF4 directly binds to the promoter of multiple key genes in the transforming growth factor-β (TGFβ) signaling pathway, thereby repressing their expression. TCF4 expression is consistently down-regulated in three EndoMT models. TCF4 down-regulation diminishes its inhibitory effect on the TGFβ signaling pathway, leading to pathway activation and subsequently enhancing EndoMT. This, in turn, further suppresses TCF4 expression. Consequently, the TCF4–TGFβ feedback loop is formed to intensify the EndoMT process. We demonstrate that introducing exogenous TCF4 disrupts this TCF4–TGFβ feedback loop of EndoMT, rescuing the EC phenotype and function under TGFβ stimulation, as well as ECs from human patients with heart failure. Our results reveal a key role for TCF4 in safeguarding EC identity and preventing EndoMT, suggesting a therapeutic potential of targeting TCF4 for EndoMT-related cardiovascular diseases.

## Graphical Abstract

**Graphical Abstract:**
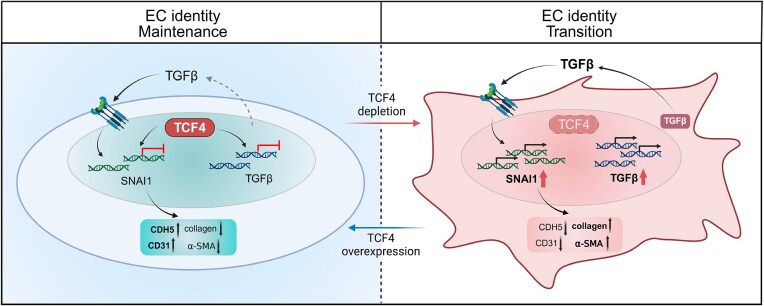

## Introduction

Endothelial cells (ECs) line the inner layer of blood vessels and are essential for maintaining the homeostasis of the circulatory system [1]. They play a central role in blood vessel formation, vascular tone control, angiogenesis, neutrophil recruitment, coagulation, and fibrinolysis [2]. ECs are characterized by remarkable plasticity [3], including their ability to undergo endothelial to mesenchymal transition (EndoMT). During EndoMT, ECs undergo a transformation in which they lose their endothelial identity and adopt mesenchymal-like characteristics, including the expression of α-smooth muscle actin (α-SMA) and the production of type I collagen. EndoMT can occur during heart development and also in many disease processes, including but not limited to cardiac [4], pulmonary [5], and kidney fibrosis [6], atherosclerosis [7], and cancer-associated fibroblast formation [8, 9]. In most cases, ECs undergo a partial EndoMT process [10–13] and exhibit both endothelial and mesenchymal traits. The resulting cells are fibrotic and produce an excessive amount of extracellular matrix, leading to tissue stiffness and impairing normal physiological functions. Numerous disease-associated factors, including members of the transforming growth factor-β (TGFβ) superfamily, interleukin-1β (IL-1β), tumor necrosis factor-α (TNF-α), high glucose levels, and endotoxins, have been shown to induce the transition of ECs into a mesenchymal-like state [14]. Conversely, physiological blood flow, particularly laminar shear stress, has been reported to support the maintenance of EC homeostasis and identity [15]. It is believed that the establishment and maintenance of EC identity require then dynamic balance of different signaling pathways [16]. However, it remains unclear what key molecular regulators control this crucial balance and prevent ECs from losing their identity.

Cellular identity is known to be established and maintained through the combinatorial function of cell identity genes [17–19]. It is increasingly recognized that genes associated with cell identity are characterized by unique epigenetic features [17, 20–22], which can be utilized to uncover cell identity genes effectively [23, 24]. Specifically, genes controlling cell identity are typically marked by a unique broad pattern of the histone modifications H3k4me3 [20, 21] and superenhancers [17, 22], in contrast to most other expressed genes marked by narrow H3k4me3 and typical enhancers. Recent work further revealed that cell identity genes tend to be regulated by a subset of superenhancers bound by CTCF (CCCTC-binding factor) [25]. Further, when compared with other expressed genes in the cell, cell identity genes tend to have the lowest RNA stability due to intense co-transcriptional m^6^A modification on the RNAs [26]. Intriguingly, those cell identity gene signatures were often also observed at tumor suppressor genes [20, 27], whereas oncogenes often display epigenetic signatures with opposite functions, e.g. the broad genic repression domains marked by widespread H3K27me3 [28].

Our current understanding of EC identity regulators and their underlying molecular mechanisms is still very limited. A comprehensive catalog of EC identity genes would greatly enhance our comprehension of EC biology and pave the way for the development of novel therapeutic strategies targeting dysregulated ECs. Although several transcription factors have been identified as regulators of EC phenotype, function, and/or differentiation, there have been very few reports of EC regulators with a documented role in regulating EC identity transition. For example, the transcription factor MECOM has been reported as a master regulator of EC identity genes [24], and is required for EC differentiation, functions, and angiogenesis [29]. The transcription factor ERG in ECs could promote vascular stability and angiogenesis through Wnt/β–catenin signaling [30]. In mature vasculature, ERG plays a crucial role in maintaining endothelial homeostasis by transactivating genes associated with essential endothelial functions and concurrently repressing the expression of pro-inflammatory genes [31]. NR2F2 exerts a critical function in determining the EC fate toward venous and lymphatic ECs [32]. However, our current understanding of the molecular mechanisms involved in regulating EC identity transition remains quite limited. Discovering EC identity regulators capable of controlling this transition will bridge the gap in our understanding of EC identity regulation and facilitate the development of novel therapeutic approaches.

Transcription factor 4 (*TCF4*, also known as *ITF2*) is a member of the basic helix–loop–helix (bHLH) protein family and should be distinguished from T-cell factor 4 (also known as TCF7L2), which is also abbreviated as TCF4 in some publications [33]. Similar to other bHLH proteins, TCF4 forms homo- or heterodimers through its bHLH domain and binds to E-box DNA elements to function as a transcriptional activator or repressor. TCF4 has been extensively studied in the context of brain development, memory, and cognition, where it plays an important role in neural lineage commitment and neuronal function [33–38]. TCF4 interacts and cooperates with SOX11 to regulate neurodevelopment by activating the expression of genes involved in axonogenesis and axon guidance [39]. In addition, TCF4 can also repress the cell adhesion molecule Neurexin, thereby limiting excessive neurite branching and synapse formation to help maintain proper neuronal circuit organization [40]. TCF4 further plays an important role in the immune system [41, 42] and has been reported to be involved in B-cell development [33]. Despite these investigations of TCF4 in other cell types, the function and underlying mechanism of TCF4 in ECs is still unknown.

In this study, through a comprehensive analysis of the endothelial epigenomic landscape combined with experimental validation, we identified *TCF4* as an EC identity gene that plays a critical role in maintaining EC phenotypes and functions. TCF4 depletion induces EC identity transition to a mesenchymal-like state. Mechanistically, we demonstrate that in normal ECs, TCF4 acts as a suppressor of the TGFβ signaling pathway by binding to the promoter regions of multiple key genes. Depletion of TCF4 in ECs relieves this suppression, leading to up-regulation of TGFβ signaling and subsequently triggering EndoMT. Conversely, overexpression of TCF4 mitigates TGFβ-induced EndoMT and rescues the dysfunction of ECs from heart failure patients. These findings establish TCF4 as a key safeguard of EC identity. The mechanistic insights uncovered in this study lay a foundation for investigating the therapeutic potential of targeting TCF4 to address EndoMT-related vascular diseases.

## Materials and methods

### Human heart sample collection from patients

This study was approved by the Institutional Review Board (IRB protocol 00 006 097) at Houston Methodist Hospital. The investigation adhered to the principles outlined in the Declaration of Helsinki. Cardiac tissue samples from heart failure patients (*n* = 8, Table 1) were de-identified and collected from clinically discarded material during left ventricular assist device (LVAD) implantation. Tissue cores were removed from the apical region of the heart, and only the midmyocardial region was sampled, with the endocardium and epicardium excised and discarded. De-identified control cardiac tissue samples (*n* = 6, Table 1) were collected from healthy donor hearts that were not used for transplantation. All the cardiac tissue used in this study was de-identified clinical waste, and a waiver of consent was approved by IRB protocol 00006097. For histology, tissues were collected and promptly fixed in 4% paraformaldehyde (PFA). They were then dehydrated in a series of graded alcohols, cleared in xylene, and embedded in paraffin using standard techniques. Sections of 5 μm thickness were cut for histological analysis. For EC isolation, tissues were dissected into small blocks and incubated in a solution of collagenase (0.5 mg/ml) for 1 h at 37°C. Subsequently, 75 μl of DNase I solution was added per 10 ml of cell suspension and incubated for another 30 min in a 37°C water bath with continuous agitation. The digested tissue was passed through a cell strainer to remove undigested blocks and then incubated for a further 10 min in 0.25% trypsin (1 ml of trypsin for every 100 mg of tissue) to obtain a single-cell suspension. ECs were then isolated using the human CD31 microbead kit (Miltenyi Biotec, Cat# 130-091-935) according to the manufacturer's instructions.

**Table 1.** Summary of de-identified human heart samples used in this study

Sample ID	Usage	Sample Information
HR 178	IHC	Control heart sample, from healthy donor without heart disease
HR 182	IHC	Control heart sample, from healthy donor without heart disease
HR 202	IHC	Control heart sample, from healthy donor without heart disease
HR 222	IHC	Control heart sample, from healthy donor without heart disease
HR 236	IHC	Control heart sample, from healthy donor without heart disease
HR 245	IHC	Control heart sample, from healthy donor without heart disease
HR 225	IHC	Heart failure sample, from LVAD implantation surgery
HR 226	IHC	Heart failure sample, from LVAD implantation surgery
HR 230	IHC	Heart failure sample, from LVAD implantation surgery
HR 246	IHC	Heart failure sample, from LVAD implantation surgery
HR 247	IHC	Heart failure sample, from LVAD implantation surgery
HR 266	IHC	Heart failure sample, from LVAD implantation surgery
HR 267	IHC	Heart failure sample, from LVAD implantation surgery
HR 293	IHC	Heart failure sample, from LVAD implantation surgery
HR 349	EC isolation	Heart failure sample, from LVAD implantation surgery
HR 351	EC isolation	Heart failure sample, from LVAD implantation surgery
HR 369	EC isolation	Heart failure sample, from LVAD implantation surgery

Immunohistochemistry (IHC); endothelial cell (EC); left ventricular assist device (LVAD).

### Cell culture

Human umbilical vein endothelial cells (HUVECs; Lonza, CC-2935) were cultured in EGM™-2 Endothelial Cell Growth Medium-2 BulletKit™ (Lonza, CC-3162) at 37°C, 5% CO_2_, and cells of passage 1–5 were used. The cell line HEK293T was purchased from the ATCC and grown in Dulbecco’s modified Eagle’s medium (DMEM) complete medium with 10% fetal bovine serum (FBS). The Platinum-A cell line was a kind gift from Dr Xianchang Li and was grown in DMEM complete medium supplemented with 10% FBS and 10 μg/ml blasticidin. All the cells were incubated at 37°C with 5% CO_2_ and continuously cultured for <2 months. Cell lines were mycoplasma negative in routine tests. Phosphate-buffered saline (PBS) (GenClone, 25-508) and Trypsin-EDTA (GenClone, 25-510) were used for cell passaging.

### Plasmids, transfection, lentiviral and retroviral production, and infection

The TCF4 short hairpin RNA (shRNA) plasmids were obtained from the MISSION shRNA library (Sigma, Supplementary Table S1). To construct the TCF4 overexpression plasmid, a DNA fragment of the TCF4-coding DNA sequence was cloned into the pMYs-IRES-GFP vector. Lentivirus was produced by co-transfection of TCF4 shRNA plasmids with third-generation lentiviral packaging plasmids pMDLg/pRRE (Addgene, #12251), pRSV-Rev (Addgene, #12253), and pMD2.G (Addgene, #12259) with Lipofectamine™ 3000 (Thermo Fisher, L3000001) into 10 cm plates with HEK293T cells. Plasmid pLKO.1 puro (Addgene, #8453) was used as control. Briefly, for each lentivirus, 70% confluent HEK293T cells were transfected with 4 μg of target plasmid, 2 μg of pMDLg, 1 μg of pMD2.G, 1 μg of pRSV-Rev, 20 μl of Lipofectamine™ 3000, and 20 μl of P3000. Fresh DMEM complete medium with 10% FBS was added to HEK293T cells 24 h after transfection. After 48 h, viral supernatants were harvested and centrifuged at 800 *g* for 10 min to pellet cell debris, and were used for infection. To make the TCF4 overexpression retrovirus, the target plasmid was transfected into Platinum-A cells by using Lipofectamine™ 3000 reagent. After the first 24 h of transfection, the medium was changed to fresh DMEM complete medium with 10% FBS and, 48 and 72 h after transfection, the supernatants were pooled, centrifuged at 800 *g* for 10 min, and used for infection. For infection, HUVECs were seeded on 6-well plates 1 day prior to transduction. The virus was supplemented with 10 μg/ml polybrene and added to cells. After 24 h, the medium was changed to EGM™-2 Endothelial Cell Growth Medium. The cells were harvested for assays 48 h after infection.

For the SNAI1 overexpression plasmid (Addgene, #115446), transfection was performed using Lipofectamine™ 3000 reagent (Thermo Fisher Scientific) according to the manufacturer's instructions. Briefly, cells were seeded in 6-well plates and cultured to reach 70–80% confluency. For each well, 2.0 μg of plasmid DNA was diluted in 125 μl of Opti-MEM™ reduced-serum medium (Thermo Fisher Scientific), followed by the addition of 4 μl of P3000 reagent. In a separate tube, 4 μl of Lipofectamine 3000 was diluted in another 125 μl of Opti-MEM. The two solutions were mixed gently and incubated at room temperature for 15 min before being added dropwise to the cells. After 6 h, the medium was replaced with fresh complete growth medium. Cells were harvested 24–48 h post-transfection for subsequent analyses.

### Immunofluorescence staining

HUVECs were seeded in a Nunc Lab-Tek II Chamber Slide™ (Thermo Fisher, 154 534). After rinsing with PBS, cells were fixed with 4% PFA for 30 min. The samples were washed with PBS and blocked with 3% bovine serum albumin (BSA) solution containing 0.1% Triton X-100 (Sigma) at 37°C for 1 h. The samples were incubated with primary antibodies including VE-cadherin (1:200, Cell Signaling Technology, 2500S), α-SMA (1:200, Cell Signaling Technology, 19245S), COL1A (1:100, Santa Cruz, sc-59772), PECAM1 (1:200, Cell Signaling Technology, 3528S), or vimentin (1:200, Cell Signaling Technology, 5741S) in 3% BSA solution at 4°C for 12 h. Then the primary antibodies were removed and washed twice with PBS. The fluorescence-conjugated secondary antibodies, including goat anti-rabbit IgG (H + L) Alexa Fluor™ Plus 594 (1:200, Invitrogen, A32740) and goat anti-mouse IgG (H + L) Alexa Fluor™ Plus 488 (1:200, Invitrogen, A32723), were applied. The slides were rinsed twice with PBS after the removal of secondary antibodies. Slides were mounted with Antifade Mounting Medium with 4′,6-diamidino-2-phenylindole (DAPI; Vector Laboratories, H-1200) and Thomas® Microscope Cover Glass Coverslips (Thermo Fisher, 1169W11). Imaging was captured with an Olympus FV3000R resonant scanning confocal microscope and analyzed with ImageJ software. Antibody information is listed in Supplementary Table S3.

### RNA isolation and reverse transcription–PCR

Total RNA was isolated from cells to generate cDNA using the RNA MiniPrep kit (Direct-zol, ZYMO Research, R2052) and amfiRivert cDNA Synthesis Platinum Master Mix (GenDEPOT, R5600-100). Each cDNA sample was amplified using Power SYBR Green PCR Master Mix (Applied Biosystems, 4367659) on the QuantStudio 6 Flex Real-time PCR System (GE Healthcare, 403115082). Briefly, the reaction conditions consisted of 2 μl of cDNA and 0.2 μM primers in a 10 μl final volume of super mix. Each cycle consisted of denaturation at 95°C for 15 s, annealing at 58.5°C for 5 s, and extension at 72°C for 10 s, respectively. Glyceraldehyde phosphate dehydrogenase (GAPDH) was used as an endogenous control to normalize each sample. The experiment was performed in triplicate with three independent experiments. The primers are listed in Supplementary Table S2.

### Western blot analysis

Proteins from cultured cells were extracted in Pierce RIPA Buffer (Thermo Fisher Scientific, 89901) with a protease and phosphatase inhibitor (Thermo Fisher Scientific, 1861280). Protein concentrations were determined by a BCA kit (Pierce, 23225). To denature proteins, lysates were added to 4× loading buffer and heated to 95°C for 10 min. Protein levels were assessed by standard sodium dodecylsulfate (SDS)–polyacrylamide gel electrophoresis (PAGE) and transferred to polyvinylidene fluoride (PVDF) membranes (BIO-RAD, 162-0177). After being blocked with 5% non-fat milk at room temperature for 1 h, the membranes were incubated with primary antibodies against TCF4 (1:2000, Abcam, ab217668), VE‐cadherin (1:1000, Cell Signaling Technology, 2500S), CD31 (1:1000, Cell Signaling Technology, 3528S), α‐SMA (1:1000, Cell Signaling Technology, 19245S), vimentin (1:1000, Cell Signaling Technology, 5741S), SNAI1 (1:1000, Cell Signaling Technology, 3879S), E-cadherin (1:1000, Cell Signaling Technology, 3195S), and GAPDH (1:5000, Santa Cruz Biotechnology, sc-32233) overnight at 4°C, followed by detection with goat anti-mouse IgG (H + L)–horseradish peroxidase (HRP; 1:5000, GenDEPOT, SA001-500), or goat anti-rabbit IgG (H + L)–HRP (1:5000, GenDEPOT, SA002-500) secondary antibody and the Clean-Blot IP Detection Kit (HRP) (Thermo Fisher Scientific, 21230). Images were captured using the ChemiDoc XRS + Molecular Imager system (BIO-RAD). Antibody information is listed in Supplementary Table S3.

### HUVEC proliferation/viability, tube formation, scratch migration, and NO measurement

To assess cell proliferation/viability, HUVECs after treatment or viral infection were seeded in a 96-well plate at a density of 3000 cells per well in endothelial growth medium (EGM). Cells were allowed to attach for 24 h and then cell proliferation/viability was monitored utilizing the CellTiter-Glo® Luminescent Cell Viability Assay (Promega, Madison, WI, USA) for 24–72 h. Results were read at 24, 48, and 72 h on the Synergy 2 Multi-Mode Reader. HUVEC tube formation and scratch migration were performed as previously described [29]. In brief, 96-well plates were coated with 50 μl of matrigel (Corning, cat. no. CB-40234A) and incubated at 37°C for 30 min. A total of 1 × 10^4^ treated HUVECs in 100 μl of EGM were seeded in each well. After 4 h, images were captured using a Leica epi-fluorescence microscope. Branch number and length were quantified using ImageJ software. Migration assay was performed by scratching the confluent HUVEC monolayer with a p200 pipette tip. Wound closure was monitored using digital photography at 0, 6, and 18 h later, and measured using the NIH Image J program. Griess assay was used for nitric oxide (NO) measurement based on the manufacturer's manual; the same number of cells was used for each group (cat. no. G-7921).

### Low-density lipoprotein uptake

Treated HUVECs were seeded in a Nunc Lab-Tek II Chamber Slide™ (Thermo Fisher, 154534) or 12-well plate. The same number of cells was used for each group. Alex Fluor 594 acetylated low-density lipoprotein (Ac-LDL; 1:100, Thermo Fisher Scientific, L35353) was added to the culture medium for the final 4 h of the incubation time. Cells were rinsed twice with PBS after removal of the medium and fixed with 4% PFA for 10 min at room temperature. Slides were mounted with Antifade Mounting Medium with DAPI (Vector Laboratories, H-1200) and Thomas® Microscope Cover Glass Coverslips (Thermo Fisher, 1169W11). Imaging was captured with an Olympus FV3000R resonant scanning confocal microscope and analyzed with ImageJ software. For flow cytometric analysis, Alexa Fluor 594 Ac-LDL-incubated HUVECs were washed, trypsinized, and centrifuged at 200 *g* for 5 min, then resuspended in FACSB-10 buffer. Fluorescence was determined using a flow cytometer (LSR II, Becton Dickinson, San Jose, CA, USA), and the data were analyzed using FlowJo software (BD Biosciences).

### CUT&RUN

CUT&RUN was performed using the SimpleChIP® Plus CUT&RUN Assay Kit (Cell Signaling Technology, #86 652) according to the manufacturer's protocol with minor modifications. Briefly, 0.5–1 × 10^6^ cells were immobilized with Concanavalin A-coated magnetic beads and permeabilized with digitonin. Samples were incubated with a primary antibody against TCF4 (Cell Signaling Technology, #46993) or SNAI1 (Cell Signaling Technology, #3879) overnight at 4°C. After washing, samples were incubated with Protein A–micrococcal nuclease (pA-MNase) for 1 h at 4°C. Digestion was initiated by adding calcium chloride and carried out on ice for 30 min. The reaction was stopped, and the released DNA fragments were purified using spin columns provided in the kit. The raw reads from CUT&RUN data of TCF4 and SNAI1 were pre-processed by the CHIPs pipeline, which includes read trimming, read mapping to the human genome (hg38), read- level quality control, and peak-level quality control. The pipeline generates the files needed for the downstream analysis, including BAM files. The BAM files with deduplicated reads were imported into DANPOS220 for genome-wide peak identification. The total sequencing reads were normalized to 25 × 10^6^, and the fragment length and extension size were set to 200 bp.

### Chromation immunoprecipitation-quantitative PCR

The ready-to-use cells were collected and washed with PBS. Protein–DNA complexes were cross‐linked using 1% formaldehyde for 10 min at room temperature, and the reaction was quenched with 125 mM glycine. Cells were washed with PBS and lysed by incubation in immunoprecipitation (IP) buffer. Chromatin was sheared by sonication (Bioruptor, Diagenode) to obtain an average size of 500–1000 bp. Protein–DNA complexes were immunoprecipitated overnight using antibodies selective for recombinant anti-TCF-4 antibody (add 2 μg to each replicate, Abcam, ab217668); normal rabbit IgG (add 2 μg to each replicate, Merck Millipore,12-370) served as negative control. The immune complexes were adsorbed with Protein A–agarose beads (Invitrogen). Immunoprecipitates were washed, eluted, and crosslinks were reversed overnight. The next day, samples were clarified by phenol:chloroform:isoamyl alcohol extraction. IP and non‐IP DNA (input) were analyzed by real‐time PCR. Primers are listed in Supplementary Table S2. Enrichment of specific promoter regions after IP was calculated as fold induction over IgG.

### RNA-seq analysis

We used TopHat v2.0.12 [43] to map the RNA-seq raw reads in FASTQ format to the hg19 human reference genome with the following parameters settings: –mate-std-dev 200 -p 8 -r 200. Using UCSC KnownGenes as reference genes, the BAM file generated by TopHat was subjected to the Cuffdiff function in Cufflink suite v2.2.1 [44] to calculate read counts and gene expression (fragments per kilobases per million, or FPKM). To identify differentially expressed genes (DEGs) between different RNA-seq samples based on read counts, we used the normalizeQuantiles, estimateCommonDisp, and estimateTagwiseDisp functions in the R package edgeR v3.14.0 to normalize the read counts, estimate common dispersion, and estimate moderated tag-wise dispersion, respectively. edgeR then defined differential genes based on a negative binomial test using a cut-off of differential false discovery rate (FDR) value <0.05 and FPKM value >1 in at least one sample.

### Chromatin immunoprecipitation-seq analysis

For ChIP-seq analysis, we used Bowtie to map ChIP-seq reads to the human reference genome version hg19, requiring a single best match for each read across the genome. We used the function Dpeak in DANPOS2 [20] to calculate the read density from the mapped reads and to define enriched peaks, with the cut-off being a Poisson test *P*-value of 1e-30 for peak calling. We set the extending length to 200 bp and bin size to 10 bp in the calculation of read density. We set the smooth width to 0 bp in order not to use any smoothing step in the calculation. The input effect was subtracted from the ChIP-seq data by DANPOS2. For the reference gene set, we downloaded the KnownGene in the GENCODE v 22 provided at the Table Browser page of the UCSC Genome Browser (http://genome.ucsc.edu/cgi-bin/hgTables). For TCF4 ChIP-seq of neuroblastoma in Supplementary Fig. S10, BAM files of the TCF4 ChIP sample (ENCFF146HUB) and IgG control sample (ENCFF160ORG) were downloaded from the ENCODE database. The obtained BAM files were imported to DANPOS2 for downstream analysis using the same parameters as mentioned above.

### Single-cell RNA-seq data analysis

For scRNA-seq of EndoMT induction using human primary ECs in Fig. 3, we requested the Seurat object of the original analysis in the previous study via the corresponding author. The cells in the control and TGFβ treatment for 7 days were used by requiring a sufficient cell number for the downstream analysis. The originally normalized gene expression and wnnUMAP coordinates were used throughout our analyses. Gene set enrichment analysis (GSEA) in scRNA-seq data was performed at the single-cell level using UCell software (version 1.3.1) [45]. Subclustering analysis was performed using the Seurat package (version 4.0.5).

For scRNA-seq of the control HUVEC sample as well as two HUVEC samples that were induced for EndoMT at day 3 and day 7 (GSE135356) [46], we downloaded the feature–barcode matrix generated by the original authors using the Cell Ranger package from 10X Genomics. We carried out analyses of processed scRNA-seq data in R version 3.5.1 with Seurat v3.1 [47]. To examine transcriptome heterogeneity and find distinct cell clusters, we performed principal component analysis (PCA) to reduce data dimensionality. For both samples, we selected the top 30 significant principal components using a permutation-based test and heuristic methods implemented in Seurat. The selected PCA loadings are used as input for graph-based cell clustering and as input for Uniform Manifold Approximation and Projection (UMAP) analysis for reduction to two dimensions for visualization purposes. To track the HUVEC cell dynamics during EndoMT, we integrate all three scRNA-seq datasets by following the Vignette of Multiple Dataset Integration and Label Transfer from the official site of Seurat (https://satijalab.org/seurat/v3.1/integration.html). The “batch-corrected” expression matrix for all pooled cells was subject to dimension reduction, cell clustering, and visualizations.

### Function enrichment analysis

We used DAVID v6.8 [48] (https://davidbioinformatics.nih.gov/tools.jsp) for Gene Ontology (GO) pathway analysis. Each term of GO Biological process with a *P*-value <0.05 is defined as significantly enriched. Functional enrichment analysis of TCF4 peaks in CUT&RUN was performed using Cistrome-GO software [49, 50] with default parameters.

### Statistical analysis

For all experimental data, at least three independent biological replicates were performed at different times. Data were analyzed using Prism 9 software (GraphPad) and are presented as means ± standard deviation (SD). The *P*-values were assessed using a two-tailed unpaired Student's *t*-test or a two-way analysis of variance (ANOVA), with *P*-values considered to be significant as follows: *P < 0.05; **P < 0.01; ***P < 0.001; ****P < 0.0001.

### Code availability

ChIP-seq and DNase-seq analyses were performed with Bowtie v1.1.0, DANPOS v2.2.3, and Multiple Experiment Viewer (MeV) v10.2. RNA-seq analyses were performed with TopHat v2.0.12, Cufflink suite v2.2.1, edgeR v3.14.0, bedtools v2.25.0, bedGraphToBigWig v4, and Genomics Viewer (IGV) v2.3.67. scRNA-seq data were processed with Seurat v2.0 installed on R version 3.5.1. Gene Ontology pathway analyses were performed with DAVID v6.8. Motif analyses were performed with HOMER v4.10. ChIP-seq peaks and motifs of TCF4 are assigned to target genes using Cumulative Analysis of Genomic Region Enrichment (CAGRE) v1.0 (https://github.com/jielv/CAGRE) and https://doi.org/10.6084/m9.figshare.30011143.v1. Two-tailed Wilcoxon test, Fisher exact test, and Student's *t*-test were performed using R v4.0.2.

## Results

### Epigenetic landscape analysis proposes *TCF4* as an EC identity gene

**Figure 1.**
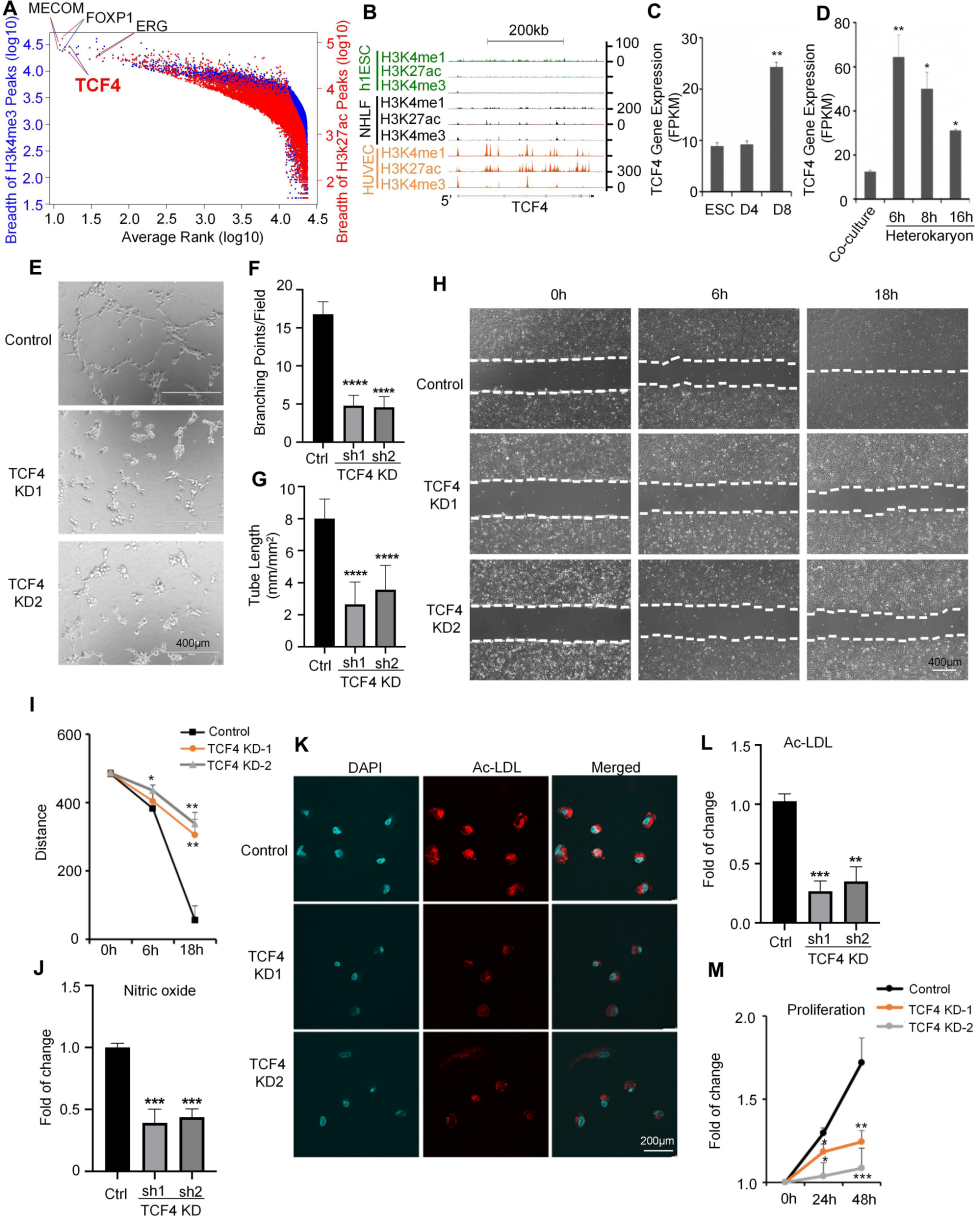
Epigenetic landscape analysis proposes *TCF4* as an EC identity gene. (**A**) Peak width of H3K4me3 and H3K27ac within ± 10 kb of the gene body of an individual gene locus in HUVECs. Ranks of each gene in terms of peak width for both markers are calculated, and genes are ordered by the sum of both ranks. (**B**) Signal density of individual chromatin marks at the *TCF4* locus in h1ESCs, HUVECs, and normal human lung fibroblasts (NHLFs). (**C** and **D**) The expression level of TCF4 at individual intervals during EC differentiation detected by RNA-seq (C), and the formation of mESC–hEC heterokaryons (**D**). (**E**) Representative images of *in vitro* tube formation in control and TCF4 knockdown (KD) HUVECs; scale bars = 400 μm. (**F**) The number of EC tube branching points per field. (**G**) The length of the EC branching tube. (**H**) Representative wound closure images at 0, 6, and 18 h after scratching; scale bars = 400 μm. (**I**) Quantification of the distance between the two borders (dotted lines in H) in wound closure images. (**J**) NO oxide production by HUVECs. (**K**) Representative images of LDL uptake; scale bars = 200 μm. (**L**) Quantification of Ac-LDL uptake. (**M**) HUVEC proliferation. The same number of cells was used for each group. Error bars represent variation between replicates. Data are presented as mean values ± SD. *n* ≥ 3 biologically independent samples. *P< 0.05, **P< 0.01, ***P< 0.001, ****P< 0.0001. *P*-values were determined by two-tailed Student's *t*-test.

To identify cell identity genes for ECs, we systematically analyzed epigenetic signatures reported to mark cell identity genes. Since the broad enrichment pattern of H3K4me3 [21] and H3K27ac [17, 22] has been reported to mark cell identity genes, we investigated the genome-wide breadth of H3K4me3 and H3K27ac at the individual gene level in HUVECs. After ranking genes based on the breadth of H3K4me3 and H3K27ac separately, we then took the average of the two rank values for each gene to generate a single rank list (Fig. 1A). Among the top-ranked transcription factors in this list, several have well-established roles in EC development and function. Notably, our analysis further revealed several top-ranked genes with few reported roles in ECs, making them promising candidates as new EC identity genes. In particular, we were highly intrigued by one of the top-ranked genes, *TCF4* (Fig. 1A). Although TCF4 has been reported to regulate neurodevelopment and dendritic cell formation [39, 40], an explicit role for TCF4 in EC biology remains unknown. DNase-seq analysis showed that the *TCF4* gene locus displays even greater chromatin openness in multiple EC subtypes compared with neurons and dendritic cells (Supplementary Fig. S1A). TCF4 mRNA abundance is also high across different EC subtypes, at similar levels or even higher compared with neurons and dendritic cells (Supplementary Fig. S1B). An extended study with public datasets of more EC subtypes confirmed that chromatin openness and mRNA abundance of TCF4 are consistently high across EC subtypes (Supplementary Fig. S1C). Manual inspection confirmed that the *TCF4* locus in HUVECs is marked by broad H3K4me3, H3K27ac, and H3K4me1 signals (Fig. 1B). In contrast, the signal of all three markers was diminished in human embryonic stem cells (h1ESCs) (Fig. 1B), suggesting *TCF4* gene activation for EC biology. Furthermore, the expression level of TCF4 increased significantly after the differentiation of ESCs into ECs (Fig. 1C). Subsequently, we studied transient heterokaryon formation between human ECs and mouse ESCs. The generation of such heterokaryons induces the expression of canonical mouse EC genes and is an approach employed to study gene regulation during EC differentiation. We analyzed TCF4 expression during transient heterokaryon formation between human ECs and mouse ESCs based on an RNA-seq dataset [51]. We observed a rapid and dramatic TCF4 up-regulation at 6 h, followed by a gradual decline as differentiation progressed, but which remained 3-fold higher than baseline after 16 h (Fig. 1D). This dynamic pattern was also seen in other key endothelial transcription factors in this model [51]. We speculate that this biphasic expression reflects an early activation phase to initiate endothelial programs, followed by sustained expression to help maintain endothelial identity. Taken together, these data propose *TCF4* as an EC identity gene.

### TCF4 is required to maintain EC phenotypes and functions

To investigate the role of TCF4 in ECs, we performed TCF4 knockdown (KD) in HUVECs using two distinct TCF4-specific shRNAs, both of which significantly reduced the mRNA and protein levels of TCF4 (Supplementary Fig. S2A, B). TCF4 KD significantly reduced the expression of Tie2 (Supplementary Fig. S2C–E), a critical EC receptor for sensing extracellular angiogenic signals and maintaining EC function. Consistently, TCF4 KD significantly impaired angiogenesis, as indicated by reduced tube formation *in vitro* (Fig. 1E). Specifically, both the number of branching points (Fig. 1F) and the tube length (Fig. 1G) were significantly decreased. TCF4 KD also impeded HUVEC migration, as evidenced by the lower rate of wound closure in TCF4 KD HUVECs using the wound scratch assay (Fig. 1H). The distance between the two edges of the wound line is significantly larger in the two TCF4 KD groups (Fig. 1I). The NO production by HUVECs was attenuated by TCF4 KD (Fig. 1J). The Ac-LDL uptake by HUVECs was also significantly decreased in response to TCF4 KD (Fig. 1K, L). Furthermore, HUVEC proliferation was significantly decreased by TCF4 KD (Fig. 1M). To verify if the other effects observed in TCF4 KD HUVECs were indirectly induced by the defective cell proliferation, we treated the HUVECs with mitomycin C to inhibit cell proliferation (Supplementary Fig. S3A). Consistent with the above results, after being treated with mitomycin C, TCF4 KD still significantly impaired EC functions, and thus these effects are not simply caused by defective cell proliferation (Supplementary Fig. S3B–G). Collectively, these data highlight the important role of TCF4 in maintaining EC functions and phenotypes.

### TCF4 depletion in ECs induces EndoMT

**Figure 2.**
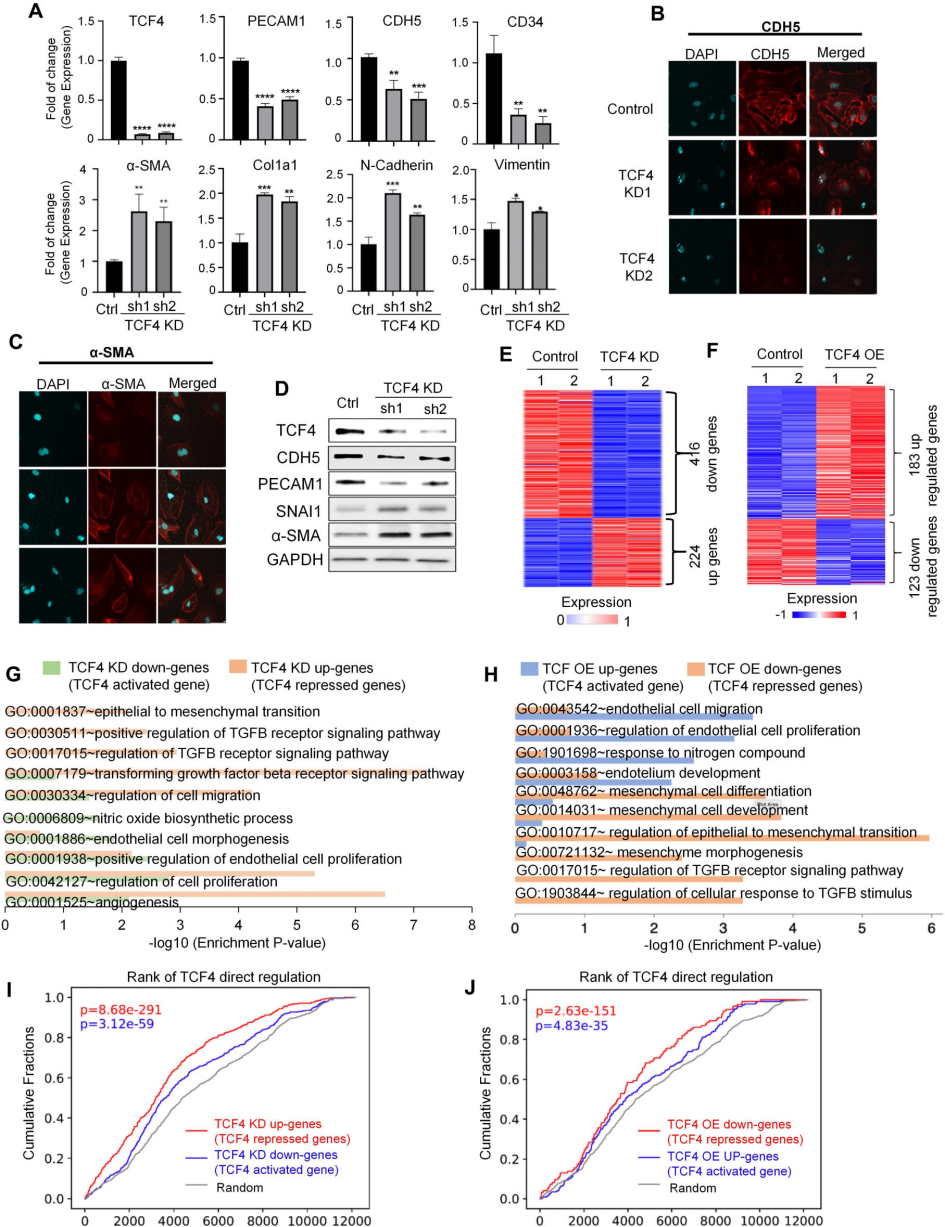
TCF4 depletion in ECs induces EndoMT. (**A**) qPCR results showing mRNA expression changes of EC markers (PECAM1, CDH5, and CD34) and mesenchymal markers (α-SMA, Col1a1, N-cadherin, and vimentin) in control and TCF4 KD HUVECs. (**B** and **C**) Representative IF staining images of CDH5 and α-SMA in control and TCF4 KD HUVECs; scale bars = 50 μm. (**D**) Western blot showing protein expression level change after TCF4 KD. (**E**) Heatmap showing DEGs between TCF4 KD and control HUVEC RNA-seq samples. Genes that pass the thresholds (FDR <0.05 and |log2foldchange| >1) are defined as either up-regulated (224) or down-regulated (416). (**F**) Heatmap showing DEGs between TCF4 overexpression (OE) and control HUVEC RNA-seq samples. (**G**) Pathways enriched in TCF4-regulated genes defined by TCF4 KD RNA-seq-based differential gene expression analysis shown in (E). (**H**) Pathways enriched in TCF4-regulated genes defined by TCF4 OE RNA-seq. (**I**) Cumulative fraction of genes plotted against TCF4 direct regulatory potential scores computed using Cistrome-GO based on TCF4 CUT&RUN peaks. Three gene sets were analyzed, naming TCF4 KD up-regulated genes (red curve), TCF4 KD down-regulated genes (blue curve), and random genes (gray curve). (**J**) Cumulative fraction of genes plotted against TCF4 direct regulatory potential scores for TCF4 OE down-regulated genes (red curve), TCF4 OE up-regulated genes (blue curve), and random genes (gray curve). Two-sided Wilcoxon rank sum test was used to compute the *P*-values. Data are presented as mean values ± SD. *n* ≥ 3 biologically independent samples. *P< 0.05, **P< 0.01, ***P< 0.001, ****P< 0.0001. *P*-values were determined by two-tailed Student's *t*-test.

As TCF4 KD significantly impaired EC phenotypes and functions, we next investigated if TCF4 KD could induce EC identity transition. We found that TCF4 KD in HUVECs significantly reduced the expression of EC marker genes, including PECAM1 (CD31), CDH5 (VE-cadherin), and CD34 (Fig. 2A). More interestingly, TCF4 KD in HUVECs further induced the expression of mesenchymal markers, such as α-SMA, COL1A1, N-cadherin, and vimentin (Fig. 2A). This observation was further confirmed by western blot and immunofluorescence (IF) staining (Fig. 2B–D; Supplementary Fig. S4A). Therefore, TCF4 KD in HUVECs induced three typical features of EndoMT, i.e. losing EC-specific markers, acquiring mesenchymal or myofibroblast phenotype, and expressing mesenchymal cell products.

To explore the underlying molecular mechanism, we performed RNA-seq for both control and TCF4 KD HUVECs. We found that TCF4 KD caused 416 down-regulated genes and 224 up-regulated genes (cut-off: FDR < 0.05 and fold change > 2) (Fig. 2E). The down-regulated genes in the TCF4 KD group (TCF4-activated genes) were enriched in EC function-related pathways, e.g. positive regulation of endothelial cell proliferation (GO:0001938), nitric oxide biosynthetic process (GO:0006809), and angiogenesis (GO:0001525). The up-regulated genes in the TCF4 KD group (TCF4-repressed genes) were enriched in pathways associated with EndoMT, e.g. TGFβ receptor signaling pathway (GO:007179) and epithelial to mesenchymal transition (EMT; a similar process to EndoMT) (GO:0001837) (Fig. 2G). In a complementary experiment, we overexpressed TCF4 in HUVECs and performed RNA-seq. We identified 183 up-regulated genes and 123 down-regulated genes by TCF4 overexpression (OE) (Fig. 2F). The TCF4-regulated transcriptional program revealed from RNA-seq data of TCF4 KD and OE HUVECs was highly consistent. TCF4 OE activates typical pathways relevant to EC functions, including endothelial cell migration (GO:0043542), endothelial cell proliferation (GO:0001936), and endothelium development (GO:0003158). TCF4 OE further repressed pathways relevant to mesenchymal functions, including mesenchymal cell differentiation (GO:0048762), epithelial to mesenchymal transition (GO:0010717), and regulation of the TGFβ receptor signaling pathway (GO:0017015) (Fig. 2H). To objectively validate our findings, we analyzed published RNA-seq datasets and identified EC- and fibroblast-specific gene sets by comparing each cell type with ESCs [52–54]. As expected, the EC-specific gene set was highly enriched for TCF4-activated genes (those down-regulated upon TCF4 KD), while the fibroblast-specific set was concurrently enriched for TCF4-repressed genes (those up-regulated upon TCF4 KD) (Supplementary Fig. S4B, C). Taken together, this transcriptome-wide evidence corroborates our conclusion that TCF4 is critical for maintaining EC function, and its depletion causes a loss of endothelial identity via EndoMT.

Given the established role of TCF4 as a transcription factor, our next inquiry was whether TCF4 represses EndoMT through direct genomic binding. To address this, we performed TCF4 CUT&RUN in HUVECs. Consistent with our RNA-seq results, GO analysis of TCF4-bound genes revealed significant enrichment in both endothelial function-related and mesenchymal function-related pathways (Supplementary Fig. S4D, E). We next assessed TCF4’s direct regulatory influence by calculating regulatory potential scores using Cistrome-GO software [49, 50]. Interestingly, DEGs from both TCF4 KD and OE experiments showed significantly higher regulatory potential than random genes (Fig. 2I, J), supporting TCF4’s dual function as both an activator and a repressor. Therefore, TCF4 is likely to prevent EndoMT by activating endothelial markers and repressing mesenchymal markers.

### scRNA-seq analysis of *in vitro* EndoMT revealed the critical role of TCF4 as a transcriptional repressor to safeguard EC identity

**Figure 3.**
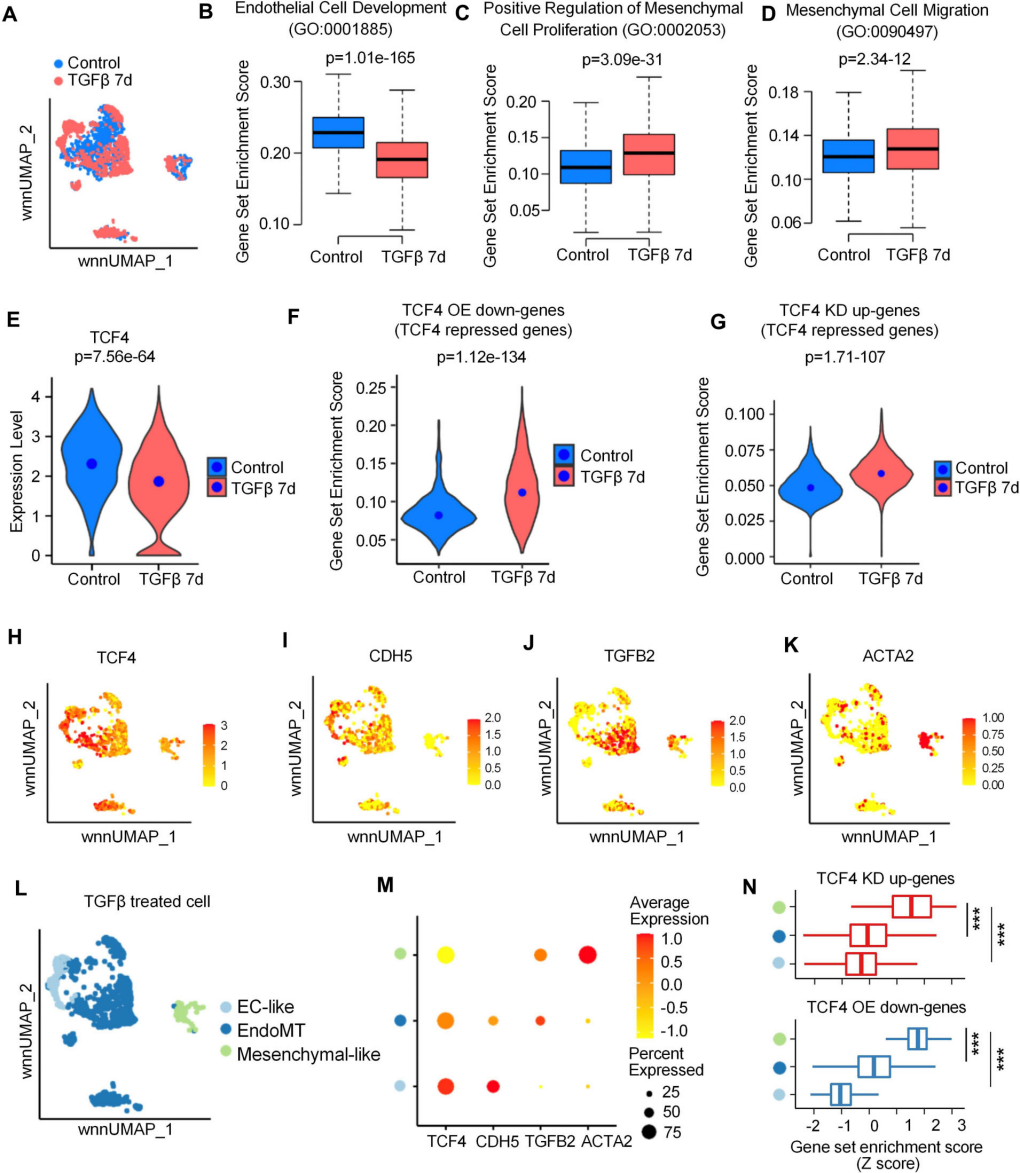
scRNA-seq analysis of *in vitro* EndoMT revealed the critical role of TCF4 as a transcriptional repressor to safeguard EC identity. (**A**) Weighted nearest-neighbor UMAP (wnnUMAP) of ECs in control (without TGFβ treatment) and TGFβ 7d (TGFβ treatment for 7 days). Each dot represents a cell. Color labels show the condition of TGFβ treatment. (**B–D**) Boxplots show the gene signature score of different gene sets in ECs of control and TGFβ 7d condition. The *y*-axis represents the gene set enrichment score computed using UCell software [45]. (B) Results for the gene set “Endothelial Cell Development”. (C) Results for the gene set “Positive Regulation of Mesenchymal Cell Proliferation”. (D) Results for the gene set “Mesenchymal Cell Migration”. Those gene sets were downloaded from the MSigDB database. (**E**) A violin plot shows the TCF4 expression level in single cells of control and TGFβ 7d conditions. The *y*-axis represents the normalized gene expression level. (**F** and **G**) Violin plots show the gene signature score of the TCF OE down-regulated gene set (F) and the TCF4 KD up-regulated gene set (G) in ECs of control and TGFβ 7d conditions. The *y*-axis represents the gene set enrichment score computed using UCell software. (**H–K**) The wnnUMAP plots show the gene expression level of TCF4, CDH5, TGFβ2, and ACTA2 genes. Each dot represents a cell. Dots are colored by normalized gene expression levels. (**L**) The wnnUMAP plot shows the EC subgroups divided into EC-like, EndoMT, and mesenchymal-like cells. Color represents EC subgroups. (**M**) Dot plot shows the averaged expression level of TCF4, CDH5, TGFβ2, and ACTA2 genes. Dot color represents the average expression level of the gene in an EC subgroup. Dot size denotes the percentage of cells expressing the gene in the cell population. (**N**) Box plots show the gene signature score of gene sets of TCF4 KD up-regulated genes and TCF4 OE down-regulated genes. The *x*-axis represents the z-score scaled gene set enrichment score computed using UCell software. The *y*-axis represents EC subgroups. Box color represents gene sets. *P*-values were computed using two-sided *t-*test for (B), (C), (D), (F), (G), and (N). *P*-values were computed using two-sided Wilcoxon test for (E). ***P-values < 0.0001.

Given the heterogeneity of ECs and the transitional nature of EndoMT, we utilized single-nucleus RNA-seq [55] data from an *in vitro* EndoMT model to investigate the heterogeneity of TCF4 expression across individual cells and its impact on the process of EndoMT. In this model, primary ECs isolated from six de-identified heart transplant donors were treated with TGFβ for 7 days to induce EndoMT (Fig. 3A). Gene signature analysis revealed that cells in the control group showed significantly higher enrichment of EC-related gene signatures compared with TGFβ-treated cells (Fig. 3B). Conversely, mesenchymal-related gene signatures were significantly enriched in the TGFβ 7d group compared with controls (Fig. 3C, D), confirming successful induction of EndoMT. We next examined how TCF4 and its target genes change during EndoMT. Interestingly, TCF4 expression was significantly down-regulated in ECs treated with TGFβ (Fig. 3E). Consistently, TCF4-repressed genes, defined as those down-regulated by TCF4 OE and up-regulated by TCF4 KD in ECs, were significantly up-regulated in the TGFβ-treated ECs (Fig. 3F, G). These findings are consistent with our observation that TCF4 represses mesenchymal pathway genes.

Further analysis of TGFβ-treated ECs revealed heterogeneous expression of TCF4 (Fig. 3H), the EC marker gene *CHD5* (Fig. 3I), as well as mesenchymal markers, including TGFβ2 and ACTA2 (Fig. 3J, K). This suggests that, despite overall induction of EndoMT, the transition remains heterogeneous at the single-cell level. To quantify this heterogeneity, we performed subclustering on ECs from the TGFβ 7d condition, classifying them into EC-like, EndoMT, and mesenchymal-like subpopulations based on marker gene expression (Fig. 3L, M). Notably, TCF4 expression showed a stepwise decrease from EC-like to EndoMT, to mesenchymal-like cells (Fig. 3M). Correspondingly, TCF4-repressed genes were expressed at significantly higher levels in mesenchymal-like cells compared with the other subpopulations (Fig. 3N). Together, these results further support the notion that TCF4 acts as a negative regulator of mesenchymal phenotypes during EndoMT.

To more comprehensively evaluate the role of TCF4 in the process of EndoMT, we analyzed an additional single-cell RNA-seq dataset [46] from an independent *in vitro* EndoMT model, in which ECs were treated with high glucose and TNFα (HT) and sampled at days 0, 3, and 7 d after treatment. Consistent with the above observations, TCF4 expression gradually decreased during EndoMT, while EC marker genes such as *CDH5*decreased and EndoMT-related genes including α-SMA and TGFβ2 increased (Supplementary Fig. S5A, B). Notably, TCF4 expression remained heterogeneous across individual cells. Cells with lower TCF4 expression (the cell population enclosed by the blue-bordered box) showed higher expression of EndoMT marker genes (Supplementary Fig. S5A), suggesting that they were more prone to transition. Further clustering analysis identified a TCF4-low cell population (C2) that decreased in proportion as EndoMT progressed (Supplementary Fig. S6A–D). Moreover, when cells were divided into TCF4-high and TCF4-low groups, the TCF4-low group exhibited greater fold changes in gene expression during EndoMT (Supplementary Fig. S5C). We then integrated this scRNA-seq dataset with our bulk RNA-seq data from TCF4 KD and OE experiments. GSEA showed that genes up-regulated during EndoMT in the scRNA-seq data significantly enrich TCF4-repressed genes from the bulk data—specifically, genes up-regulated by TCF4 KD and down-regulated by TCF4 OE (Supplementary Fig. S5D). By contrast, TCF4-activated genes showed no significant enrichment of genes altered during EndoMT (Supplementary Fig. S5D). Additionally, genes positively correlated with TCF4 expression in control ECs were significantly enriched among TCF4-activated genes identified in bulk RNA-seq (Supplementary Fig. S5E). In contrast, TCF4-repressed genes were not significantly enriched in genes correlated with TCF4 expression (Supplementary Fig. S5E). These results suggest that TCF4 maintains normal endothelial homeostasis by activating numerous genes, while it simultaneously acts as a transcriptional repressor to specifically inhibit the EndoMT process, thereby safeguarding EC identity.

Taken together, these results reveal that the expression level of TCF4 plays a critical role in maintaining EC identity, whereas its expression heterogeneity directly influences the extent of EndoMT.

### scRNA-seq analysis of lineage tracing in mice indicates TCF4 up-regulation in mesenchymal to endothelial transition

As TCF4 KD could induce EndoMT, we next investigated the expression change of TCF4 during mesenchymal to endothelial transition (MEndoT), which is the reverse process of EndoMT and is involved in angiogenesis and vasculogenesis [56, 57]. To this end, we analyzed our recent scRNA-seq data from Fsp1-Cre:R26R-EYFP fibroblast lineage tracing in the hindlimb ischemia mice model [58]. The data were generated using isolated yellow fluorescent protein (YFP)+ CD11b– fibroblasts on day 0 and day 28 (recovery stage) after femoral artery ligation. We identified 11 clusters of YFP+ CD11b– cells (Supplementary Fig. S7A) based on single-cell-level transcriptome similarity. We found that cluster 0 is the cell population undergoing MEndoT, as indicated by the expression of the EC-specific marker CDH5 (Supplementary Fig. S7B). Interestingly, TCF4 also showed the highest RNA expression level in cluster 0 compared with other clusters on day 28 (Supplementary Fig. S7C, D). The percentage of cluster 0 cells in the sample increased from day 0 to day 28 (Supplementary Fig. S7E). Furthermore, the TCF4 reads count in cluster 0, as well as the proportion of TCF4-positive ECs, also showed a marked increase on day 28 (Supplementary Fig. S7F, G). These data indicated that the TCF4 expression level is increased during the process of MEndoT *in vivo*. We further analyzed bulk RNA-seq data during *in vitro* conversion of human fibroblasts to functional ECs by defined factors [59]. Consistent with the results from *in vivo* MEndoT, the TCF4 gene expression level is also highly increased in fibroblast-derived ECs and primary ECs (HUVECs) compared with fibroblasts (Supplementary Fig. S7H). We next compared available published data from different subtypes of ECs, fibroblasts, and stem cells. DNase-seq analysis showed that the *TCF4* gene locus had stronger chromatin openness in different EC subtypes compared with fibroblasts and stem cells (Supplementary Fig. S8A). Moreover, the TCF4 mRNA expression level is significantly higher across different EC subtypes compared with fibroblasts and stem cells (Supplementary Fig. S8B). Collectively, these data indicate an up-regulation of TCF4 in MEndoT.

### TCF4 modulates the TGFβ signaling pathway in EndoMT

**Figure 4.**
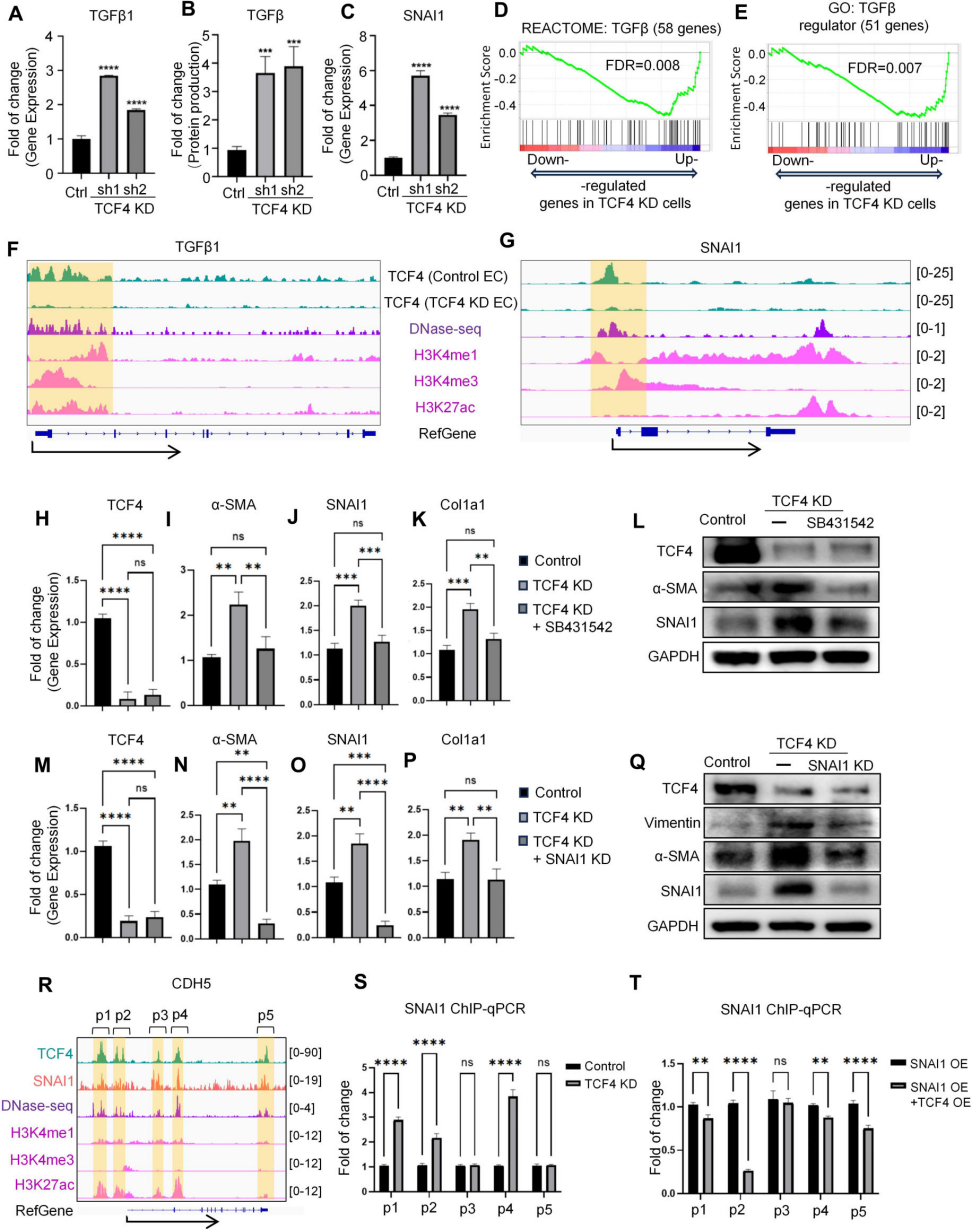
TCF4 modulates the TGFβ signaling pathway in EndoMT. (**A**) qPCR results showing gene expression changes of TGFβ1 after TCF4 KD in HUVECs. (**B**) ELISA results showing protein expression changes of TGFβ after TCF4 KD in HUVECs. (**C**) qPCR results showing gene expression changes of SNAI1 after TCF4 KD in HUVECs. (**D** and **E**) GSEA of the gene signature on differential gene expression data from TCF4 KD versus control RNA-seq. Results for gene sets of TGFβ signaling pathways from two different sources are shown. (**F** and**G**) Genomic tracks showing TCF4 peaks at TGFβ1 gene loci (F) and at SNAI1 gene loci (G) (highlighted in orange rectangles), along with other epigenetic signals, including DNase-seq, H3K4me1, H3K4me1, and H3K27ac. (**H–K**) qPCR results showing mRNA expression changes of TCF4 and mesenchymal markers (α-SMA, SNAI1, and Col1a1) in control, TCF4 KD, and TCF4 KD + SB431542 HUVECs. (**L**) Western blot showing changes in protein expression levels after TCF4 KD and SB431542 treatment. (**M–P**) qPCR results showing mRNA expression changes of TCF4 and mesenchymal markers (α-SMA, SNAI1, and Col1a1) in control, TCF4 KD, and TCF4 KD + SNAI1 KD HUVECs. (**Q**) Western blot showing protein expression level change after TCF4 KD and SNAI1 KD. (**R**) Genomic tracks of CUT&RUN showing the binding of TCF4 and SNAI1 at the *CDH5* gene region (highlighted in orange rectangles), along with other tracks of DNase-seq and related histone modification. (**S**) SNAI1 ChIP-qPCR in both control and TCF4 KD HUVECs for the SNAI1–CDH5 binding site. (**T**) SNAI1 ChIP-qPCR in both SNAI1 OE and TCF4 OE HUVECs for the SNAI1–CDH5 binding site *P< 0.05, **P< 0.01, ***P< 0.001, ****P< 0.0001. *P*-values were determined by two-tailed Student's *t*-test.

According to our RNA-seq data in HUVECs with TCF4 KD and OE, genes repressed by TCF4 were highly enriched in the TGFβ signaling pathway (Fig. 2G, H). We next investigated if TCF4 inhibits the TGFβ signaling pathway, a key pathway in EndoMT. qPCR showed that TCF4 KD in HUVECs increased the expression of several TGFβ pathway activators, e.g. TGFβ1 (Fig. 4A), SNAI1 (Fig. 4C), TGFβR1 (Supplementary Fig. S9A), and SNAI2 (Supplementary Fig. S9B). Increased TGFβ and SNAI1 protein levels were further confirmed by enzyme-linked immunosorbent assay (ELISA) (Fig. 4B) and western blot (Fig. 4L, Q). Meanwhile, TCF4 knockdown led to an increase in SMAD2/3 phosphorylation, suggesting an enhanced activation of the TGFβ signaling pathway (Supplementary Fig. S9C). GSEA using multiple databases revealed that the TCF4-repressed genes (up-regulated by TCF4 KD in HUVECs) are significantly enriched in the TGFβ pathway, supporting a repressive role for TCF4 on this pathway (Fig. 4D, E; Supplementary Fig. S9D, E). To investigate whether this repression is due to direct binding of TCF4, we performed CUT&RUN for TCF4 in HUVECs and found that TCF4 shows EC-specific binding compared with other cell types, such as neurons (Supplementary Fig. S10). We identified several TCF4-binding peaks near the promoter region of SNAI1, TGFβ1, TGFβR1, and SNAI2 (highlighted in orange color, Fig. 4F, G; Supplementary Fig. S9F, G), which were not observed upon TCF4 KD. These peaks overlapped with strong DNase-seq signals in HUVECs, indicating accessible chromatin regions. ChIP-qPCR further validated these binding events (Supplementary Fig. S9H–J). Together, these results suggest that TCF4 represses the TGFβ signaling pathway by directly binding to the promoters of key pathway activators.

Next, we examined the functional importance of TCF4-mediated inhibition of the TGFβ pathway in the suppression of EndoMT. Our results showed that treatment with the TGFβ receptor inhibitor SB431542 effectively suppressed TCF4 deletion-induced EndoMT, as indicated by reduced expression of α-SMA, SNAI1, and Col1a1 (Fig. 4H–L; Supplementary Fig. S11D, E). Similarly, KD of SNAI1 also attenuated EndoMT triggered by TCF4 deletion, reflected by decreased α-SMA and Col1a1 levels (Fig. 4M–Q; Supplementary Fig. S12D, E). These findings were confirmed by qPCR, western blot, and IF staining. Moreover, while SNAI1 overexpression enhanced the expression of mesenchymal markers, this effect was effectively reversed by TCF4 overexpression (Supplementary Fig. S12F). Interestingly, inhibition of the TGFβ pathway did not rescue the reduction of EC marker genes caused by TCF4 deletion (Supplementary Figs S11A–E and S12A–E), suggesting that TCF4 might maintain endothelial identity further through broader transcriptional regulation beyond repressing the TGFβ pathway.

Furthermore, analysis of TCF4 and SNAI1 CUT&RUN sequencing data in HUVECs revealed substantial overlap in their binding peaks, including at the CDH5 locus (Fig. 4R). To test whether TCF4 and SNAI1 might compete for binding at these sites, we selected five regions within the *CDH5* gene bound by both proteins (Fig. 4R). ChIP-qPCR showed that TCF4 KD increased SNAI1 binding at three of these shared regions (Fig. 4S), while TCF4 overexpression reduced SNAI1 binding at four regions (Fig. 4T). These results suggest that TCF4 may also inhibit the TGFβ pathway by competing with SNAI1 for binding to key regulatory sites.

### TCF4 overexpression alleviates the TGFβ-induced EndoMT

**Figure 5.**
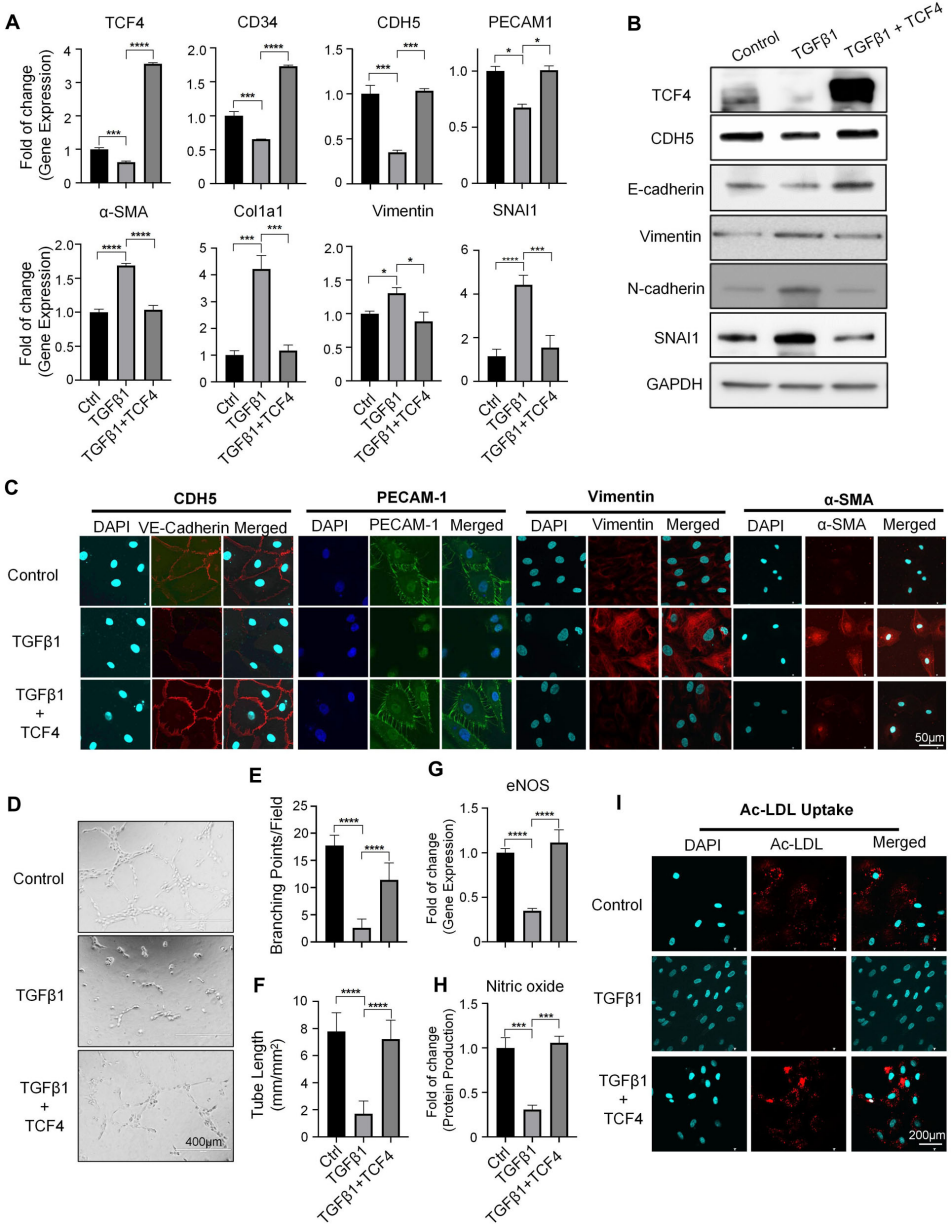
TCF4 overexpression alleviates TGFβ-induced EndoMT. (**A–C**) qPCR results (A) showing gene expression level changes, western blot (B) and IF staining (C) showing protein expression level change, after TGFβ1 treatment with or without TCF4 OE; scale bars = 50 μm. (**D**) Representative images of *in vitro* tube formation; scale bars = 400 μm. (**E**) The number of EC tube branching points per field. (**F**) The length of EC branching tubes. (**G**) qPCR results of eNOS. (**H**) NO production. (**I**) Images of Ac-LDL uptake; scale bars = 200 μm. Error bars represent variation between replicates. Data are presented as mean values ± SD. *n*≥ 3 biologically independent samples. *P< 0.05, **P< 0.01, ***P< 0.001, ****P< 0.0001. *P*-values were determined by two-tailed Student's *t-t*est.

To further verify our observation that TCF4 is down-regulated during the process of EndoMT (Fig. 3E), we next treated HUVECs with different doses of TGFβ. We found that both the gene and protein expression levels of TCF4 decreased in a dose- and time-dependent manner in response to TGFβ treatment (Supplementary Figs S13A, B, and S14A). Meanwhile, the expression level of mesenchymal marker genes, such as α-SMA, collagen, SNAI2, and vimentin, increased over time (Supplementary Figs S13A, B and S14A, B), which indicates the extent of EndoMT. We next asked if TCF4 OE could alleviate the TGFβ-induced EndoMT. Using HUVECs treated with 10 ng/ml TGFβ as a model, we observed that the cells showed significantly decreased expression of EC markers genes (CDH5, PECAM1, and CD34) and increased expression of mesenchymal marker genes (α-SMA, COL1A1, cimentin, and SNAI1) on the mRNA and protein levels (Fig. 5A–C). Remarkably, TCF4 OE in HUVECs during TGFβ treatment could help HUVECs maintain EC identity gene expression and alleviate TGFβ-induced EndoMT gene expression (Fig. 5A–C; Supplementary Fig. S14). Furthermore, TCF4 overexpression in HUVECs could maintain EC-specific functions, such as angiogenesis (indicated by tube formation assay in Fig. 5D–F), endothelial nitric oxide synthase (eNOS) gene expression (Fig. 5G), NO production (Fig. 5H), and Ac-LDL uptake (Fig. 5I).

### Exogenous TCF4 expression mitigates EndoMT and EC dysfunction in ECs from patients with hypertension-associated heart failure

**Figure 6.**
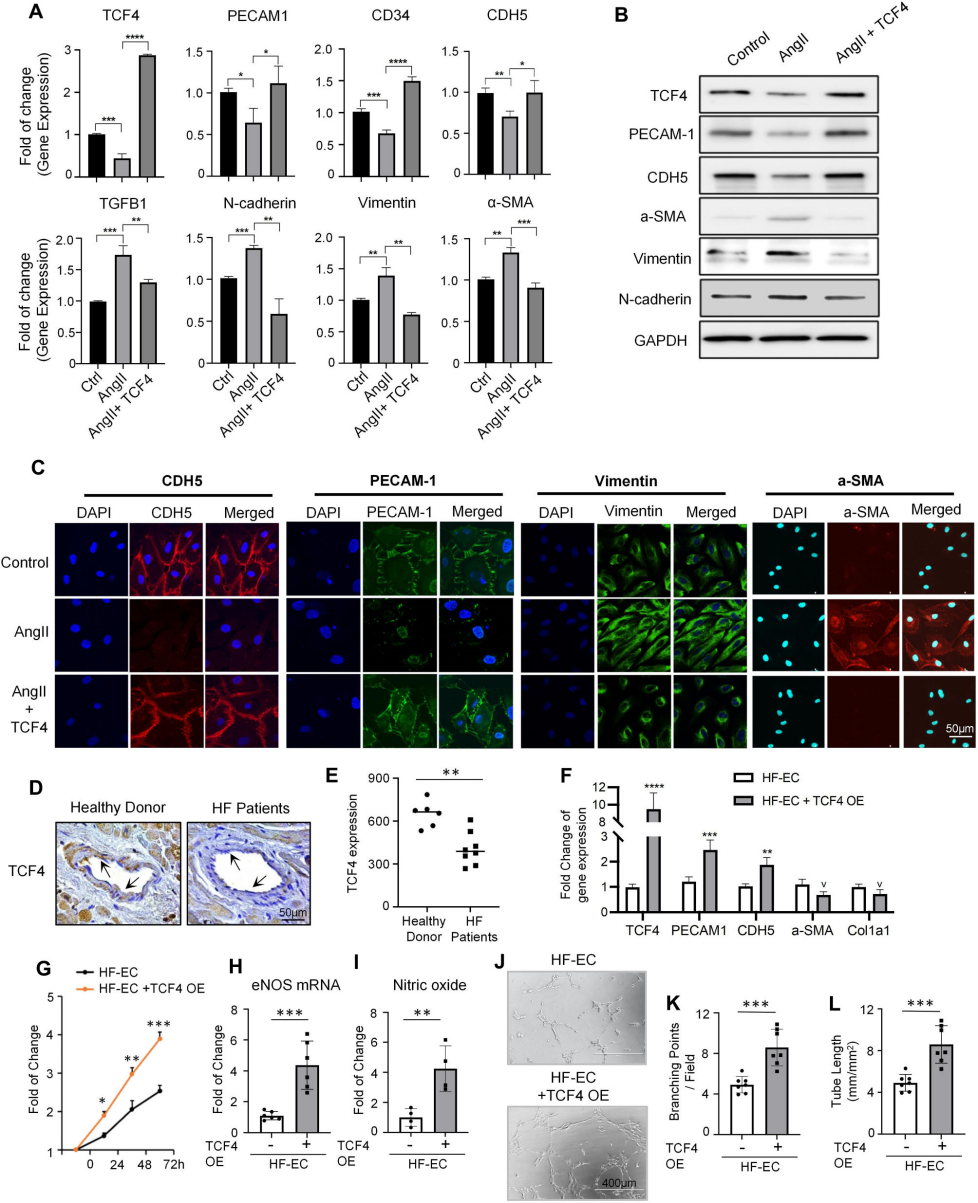
Overexpression of TCF4 mitigates EndoMT and EC dysfunction in ECs isolated from patients with hypertension-associated heart failure. (**A–C**) qPCR results (A) showing gene expression level changes, western blot (B) and IF staining (C) showing protein expression level change, after AngII treatment with or without TCF4 OE. Scale bars = 50 μm. (**D**) Representative images of TCF4 staining. The black arrow indicates a vascular EC. Scale bars = 50 μm. (**E**) Quantification of TCF4 expression in ECs. (**F–I**) Changes in gene expression (F), cell proliferation rate (G), eNOS mRNA expression level (H), and NO production (I) after TCF4 OE in isolated ECs from heart failure patients. (**J–L**) Representative images of *in vitro* tube formation; scale bars = 400 μm (J), the number of EC tube branching points per field (K), and the length of EC branching tubes (L) in isolated ECs from heart failure patients with or without TCF4 OE. Error bars represent variation between replicates. Data are presented as mean values ± SD. *n* ≥ 3 biologically independent samples. *P< 0.05, **P< 0.01, ***P< 0.001, ****P< 0.0001. *P*-values were determined by two-tailed Student's *t*-test.

Genetic lineage tracing studies have shown that EndoMT is not the major contributor to interstitial fibrosis in heart failure [60, 61]. Instead, many subsequent studies have revealed that EndoMT contributes to EC dysfunction in pulmonary arterial hypertension and hypertension-associated heart failure [62–64]. To further investigate the role of TCF4 in safeguarding EC function and inhibiting EndoMT, we treated human primary microvascular ECs with angiotensin II (AngII). Increased serum levels of AngII have been reported in large groups of patients with hypertension-associated cardiovascular diseases, and have been proven to be potent pro-fibrotic molecules by activating the TGFβ signaling pathway and exacerbating EndoMT [65, 66]. We found that both the gene and protein expression levels of TCF4 were significantly decreased in response to AngII treatment (Fig. 6A, B). Meanwhile, AngII-treated ECs showed significantly reduced expression of EC marker genes and increased expression of mesenchymal marker genes on both mRNA and protein levels (Fig. 6A–C). Importantly, TCF4 overexpression in ECs during AngII treatment could help maintain EC identity and alleviate AngII-induced EndoMT by restoring the expression of EC marker genes (PECAM1, CD34, and CDH5) and reducing expression of mesenchymal marker genes (N-cadherin, α-SMA, and vimentin) on mRNA and protein levels (Fig. 6A–C).

To further validate our findings, we obtained eight cardiac tissue samples from patients with hypertension-associated heart failure at the time of LVAD implantation, as well as six samples from normal donor hearts that were not implanted. Immunohistochemistry staining showed that TCF4 was significantly down-regulated in microvasculature ECs of heart failure patients, compared with normal donor controls (Fig. 6D, E). To further test if exogenous TCF4 expression could mitigate EndoMT and EC dysfunction in heart failure patients, we isolated ECs from the tissue of heart failure patients. qPCR results show that TCF4 OE increases the gene expression of EC marker genes (PECAM1 and CDH5) and reduces the expression of mesenchymal marker genes (α-SMA and COL1A1) (Fig. 6F). Furthermore, TCF4 overexpression in ECs of heart failure patients increases their proliferation rate (Fig. 6G), and up-regulates their EC-specific functions, such as enhanced eNOS gene expression (Fig. 6H), increased NO production (Fig. 6I), and promotion of angiogenesis (as indicated by tube formation assay in Fig. 6J–L). These data suggest that TCF4 plays a crucial role in maintaining EC identity. Furthermore, TCF4 OE could alleviate EndoMT and dysfunction of ECs derived from patients with hypertension-associated heart failure.

## Discussion

The precise regulation of the cell identity genes is critical for maintaining cell phenotype and function. The current understanding of the molecular program governing EC identity is still very limited. To address this knowledge gap, we integrated a large volume of epigenomic data derived from ChIP-seq and DNase-seq along with scRNA-seq from lineage tracing in mice and *in vitro* EndoMT models to identify cell identity genes, followed by extensive experimental investigations. Through these approaches, we discovered that *TCF4* is an EC identity gene critical for maintaining the phenotype and function of vascular ECs. We further provide evidence that EndoMT can be triggered by the loss of TCF4, which activates EC identity genes and suppresses genes involved in EndoMT.

TCF4 has been recognized for its significant involvement in various cell types, including neurons, B cells, and plasmacytoid dendritic cells (pDCs), highlighting its essential role in multiple biological contexts. Interestingly, a genome-wide association study (GWAS) revealed the presence of common polymorphisms near the *TCF4* gene, establishing a clear link between these variants and an elevated risk of developing Fuchs endothelial corneal dystrophy (FECD) [67]. This association has been consistently observed across diverse patient populations in independent studies [68–70]. It is important to note that although both corneal endothelial cells and vascular endothelial cells share the name “endothelial cell”, they exhibit significant differences in their developmental origins as well as functional phenotypes. Corneal ECs are derived from the neural crest during embryogenesis and have a neuroepithelial origin [71, 72]. In contrast, vascular ECs arise from the mesoderm [73] and possess robust regenerative capabilities [74], actively participating in angiogenesis and vasculogenesis. Corneal ECs lack inherent regenerative potential *in vivo* and require lifelong maintenance [75]. Therefore, the role of TCF4 in vascular ECs is largely unknown and requires further investigation.

Our study uncovered the significant role of TCF4 as a pivotal regulator of EC identity. We observed that knocking down TCF4 in ECs led to substantial impairments in both their phenotype and function. Intriguingly, TCF4 depletion induced EndoMT in ECs. Furthermore, single-cell transcriptome analysis revealed an association between TCF4 expression levels and the extent of EndoMT in different EC subpopulations. Specifically, the EC subpopulation with lower *TCF4* gene expression exhibited higher expression of EndoMT marker genes during the process of EndoMT, while higher TCF4 expression in the EC subpopulation protects the EC from EndoMT. These findings emphasize the critical role of TCF4 in preserving EC identity and safeguarding against the loss of EC phenotype and function, thereby preventing their transition into alternative cell types, such as mesenchymal cells.

The TGFβ signaling pathway is a canonical driver of EndoMT [14]. Operating through both SMAD-dependent and SMAD-independent cascades, TGFβ orchestrates this phenotypic switch by activating a core set of transcriptional effectors, most prominently among them the Snail family of zinc-finger proteins (SNAI1 and SNAI2) [76]. These factors execute the EndoMT program via a dual mechanism: they directly repress key endothelial identity genes, such as *CDH5*, by binding to their promoters, while concurrently activating a mesenchymal gene expression program that includes α-SMA and fibronectin [77]. In this capacity, SNAI1 acts as a pivotal molecular switch, translating upstream TGFβ signals into the profound cellular reprogramming that defines EndoMT. In this context, our study reveals that TCF4 safeguards ECs from EndoMT through the suppression of the TGFβ signaling pathway. Based on RNA-seq data in TCF4 KD and OE HUVECs, we observed a significant enrichment of the TGFβ signaling pathway and EMT genes among the repressed targets of TCF4. Additionally, our CUT&RUN sequencing and ChIP-qPCR experiments revealed that TCF4 directly binds to the promoter regions of multiple key genes in the TGFβ pathway, leading to the repression of their expression. The involvement of TCF4 in regulating EndoMT has not been previously reported. Therefore, this study highlights an important role for TCF4 in safeguarding EC identity and protecting ECs against EndoMT by inhibiting the TGFβ signaling pathway.

EndoMT is a significant contributor to EC dysfunction observed in various cardiovascular diseases. The induction of EndoMT can occur due to factors such as inflammation, oxidative stress, metabolic alterations, hypoxia, and disturbed shear stress [78]. In this study, we employed three distinct *in vitro* models to investigate EndoMT, namely TGFβ1 stimulation, high-glucose/TNFα treatment, and AngII stimulation. Our findings revealed a significant and consistent down-regulation of TCF4 expression across all these EndoMT models, suggesting that the disruption of TCF4 and its regulated signaling network is an imperative mechanism underlying EndoMT. This down-regulation of TCF4 further decreased its inhibitory effect on the TGFβ signaling pathway, resulting in increased secretion of TGFβ1 by ECs, one of the most crucial mediators of EndoMT [14, 77]. As a result, a positive feedback loop for EndoMT formed in the TCF4-depleted ECs, intensifying the process of EndoMT. Notably, our results also demonstrated that exogenous TCF4 supplementation could disrupt this feedback loop, rescuing the phenotype and function of ECs under TGFβ stimulation, as well as ECs from heart failure patients. These findings strongly suggest that TCF4 acts as a key modulator of EC fate and EndoMT. The ability of TCF4 supplementation to counteract the EndoMT feedback loop and restore EC phenotype and function supports a potential to develop therapeutic strategies to curb EndoMT and intervene in various disease conditions that involve EndoMT.

## Supplementary Material

gkaf931_Supplemental_File

## Data Availability

RNA-seq data of control, TCF4 knockdown, and TCF4 overexpression in HUVECs have been submitted to the NCBI Gene Expression Omnibus (GEO; https://www.ncbi.nlm.nih.gov/geo) under accession number GSE242102. CUT&RUN data for TCF4 and SNAI1 in HUVECs have been submitted to the NCBI GEO under accession number GSE302125. The H3K4me3, H3K27ac, and H3K4me1 histone modification ChIP-seq, DNase-seq, and RNA-seq for HUVECs, H1-hESCs, and NHLFs are downloaded from the ENCODE project website [52]. scRNA-seq data of HUVECs treated for EndoMT are downloaded from the GEO (GSE135356) [46].

## Supplementary data

Supplementary data are available at NAR online.
