# Correction to ‘Transcription factor 4 maintains endothelial cell identity by inhibiting endothelial to mesenchymal transition’

**DOI:** 10.1093/nar/gkag456

**Published:** 2026-05-05

This is a correction to: Gaopeng Xian, Rongbin Zheng, Jie Lv, Sen Zhu, Min Chen, Xinlei Gao, Shenli Yuan, Zhen Bouman Chen, Keith Youker, John P Cooke, Kaifu Chen, Lili Zhang, Transcription factor 4 maintains endothelial cell identity by inhibiting endothelial to mesenchymal transition, *Nucleic Acids Research*, Volume 53, Issue 20, 11 November 2025, gkaf931, https://doi.org/10.1093/nar/gkaf931

In the Materials and Methods section of the originally published article, the TCF4 antibody catalog number for the CUT&RUN experiments was incorrectly listed as #2569.

The catalog number has been corrected to read #46993.

