# Supplementary material for: Transcription factor 4 maintains endothelial cell identity by inhibiting endothelial to mesenchymal transition
Source: Nucleic Acids Res. 2025 Oct 29;53(20):gkaf931. doi: 10.1093/nar/gkaf931 (PMC12571441; doi:10.1093/nar/gkaf931)
Supplement: gkaf931_Supplemental_File [file gkaf931_supplemental_file.pdf]

**Supplementary tables and Figures**
**(Fig.S1-S14, Table.S1-S3)**

# Transcription Factor 4 Maintains Endothelial Cell Identity by Inhibiting Endothelial to Mesenchymal Transition

Gaopeng Xian[1,2,6], Rongbin Zheng[1,2,6], Jie Lv[3,6], Sen Zhu[3] , Min Chen[1,2], Xinlei Gao[1,2], Shenli Yuan[1,2], Zhen Bouman Chen[4], Keith Youker[5], John P. Cooke[5], Kaifu Chen[1,2,3,5,^], Lili Zhang[1,2,5,^]

**Short Title**: TCF4 Inhibits EndoMT to Preserve Endothelial Identity

[1] Basic and Translational Research Division, Department of Cardiology, Boston Children's Hospital, Boston, MA, USA
[2] Department of Pediatrics, Harvard Medical School, Boston, MA, USA
[3] Center for Bioinformatics and Computational Biology, Department of Cardiovascular Sciences, Houston Methodist Research Institute, Houston, TX, USA
[4] Department of Diabetes Complications and Metabolism, City of Hope, Duarte, CA.
[5] Center for Cardiovascular Regeneration, Department of Cardiovascular Sciences, Houston Methodist Research Institute, Houston, TX, USA
[6] Contributed equally

^ Corresponding:
Lili Zhang, Lili.Zhang@childrens.harvard.edu
Kaifu Chen, Kaifu.chen@childrens.harvard.edu

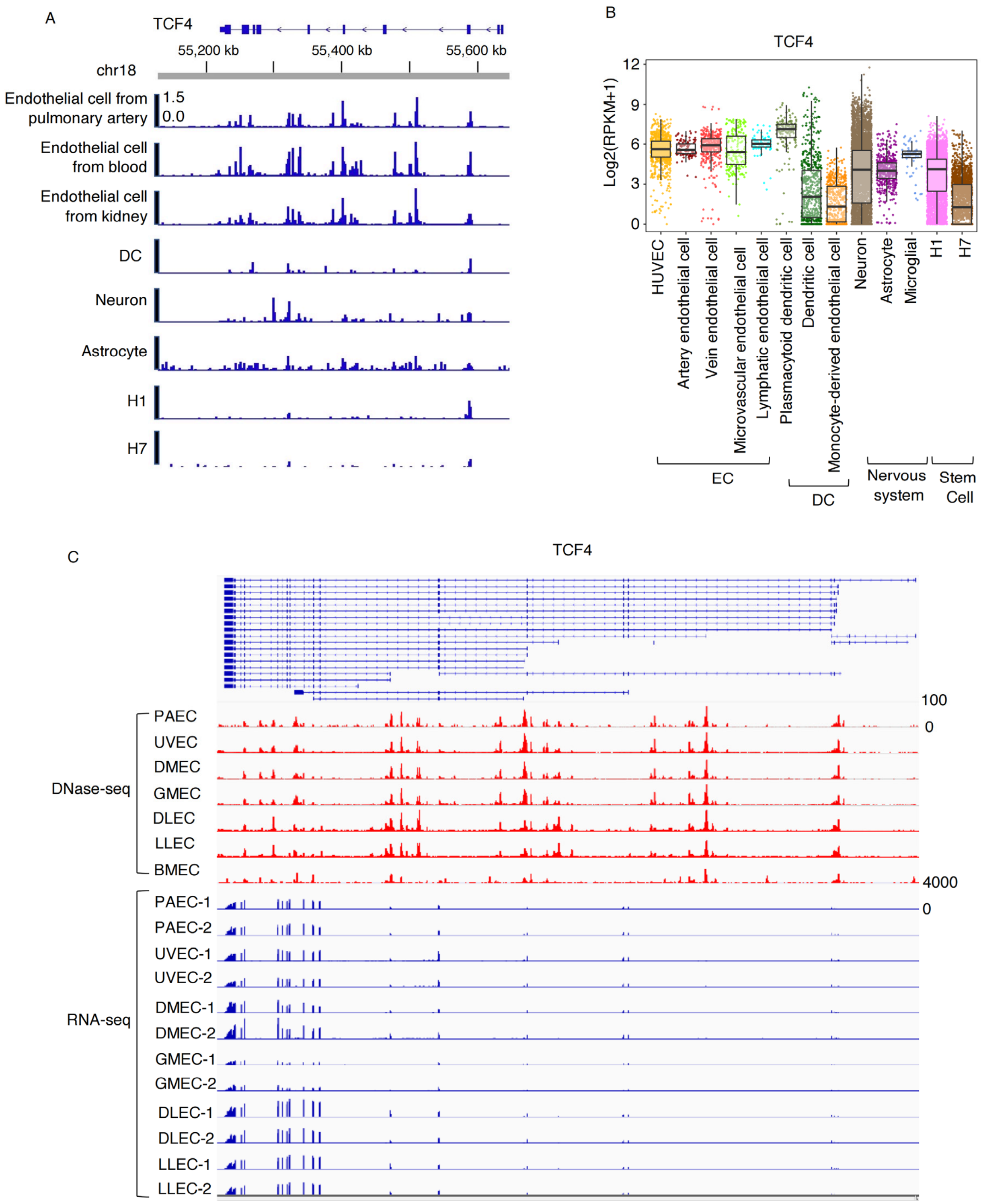


**Figure s1 Epigenetic landscape suggests TCF4 is a cell identity gene for EC**

(**A**) The representative WashU epigenome tracks of DNase-Seq profiles showed the chromatin accessibility at the TCF4 gene promoter across different cell types, including EC subtypes, dendritic cells, neural cells, and stem cells. (**B**) The gene expression of TCF4 in RNA-Seq profiles of different cell types. Each dot in the box plot represents one RNA-Seq sample obtained from ARCHS4 project. Box plots indicate median (middle line), 25th, 75th percentile (box), and

5th and 95th percentile (whiskers). (**C**) Genome tracks of DNase-Seq and RNA-Seq signal at TCF4 loci across different endothelial cell subtypes.

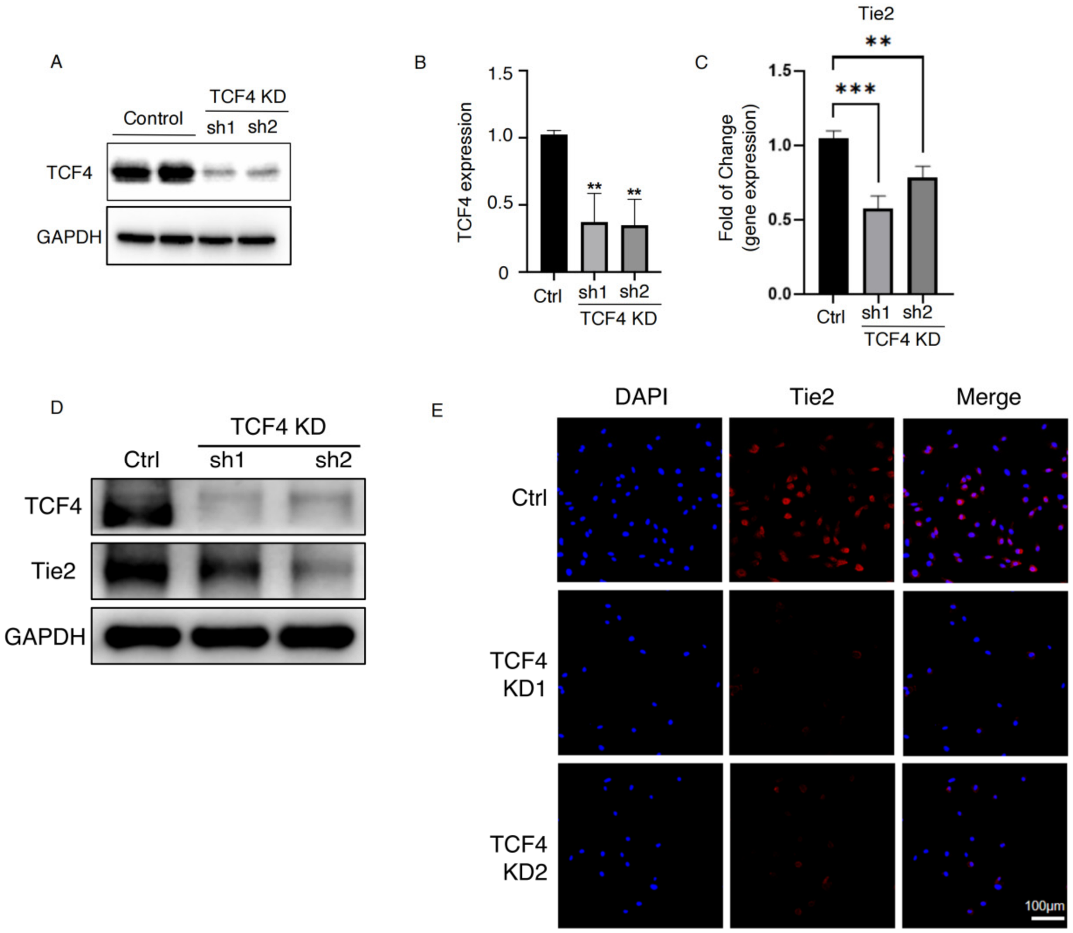


**Figure s2 TCF4 KD reduce Tie2 expression in EC**

(**A**) Western-blot results showing TCF4 protein production changes after TCF4 KD. (**B**) qPCR results showing TCF4 gene expression changes after TCF4 KD. (**C-E**) qPCR (C), western blot (D), and immunofluorescence staining (E) showing Tie2 expression level change after TCF4 KD, scale bars 100 μm. Data are presented as mean values +/- SD. n≥3 biologically independent samples. *P<0.05, **P<0.01, ***P<0.001, ****P<0.0001. P values determined by two-tailed Student's t-test.

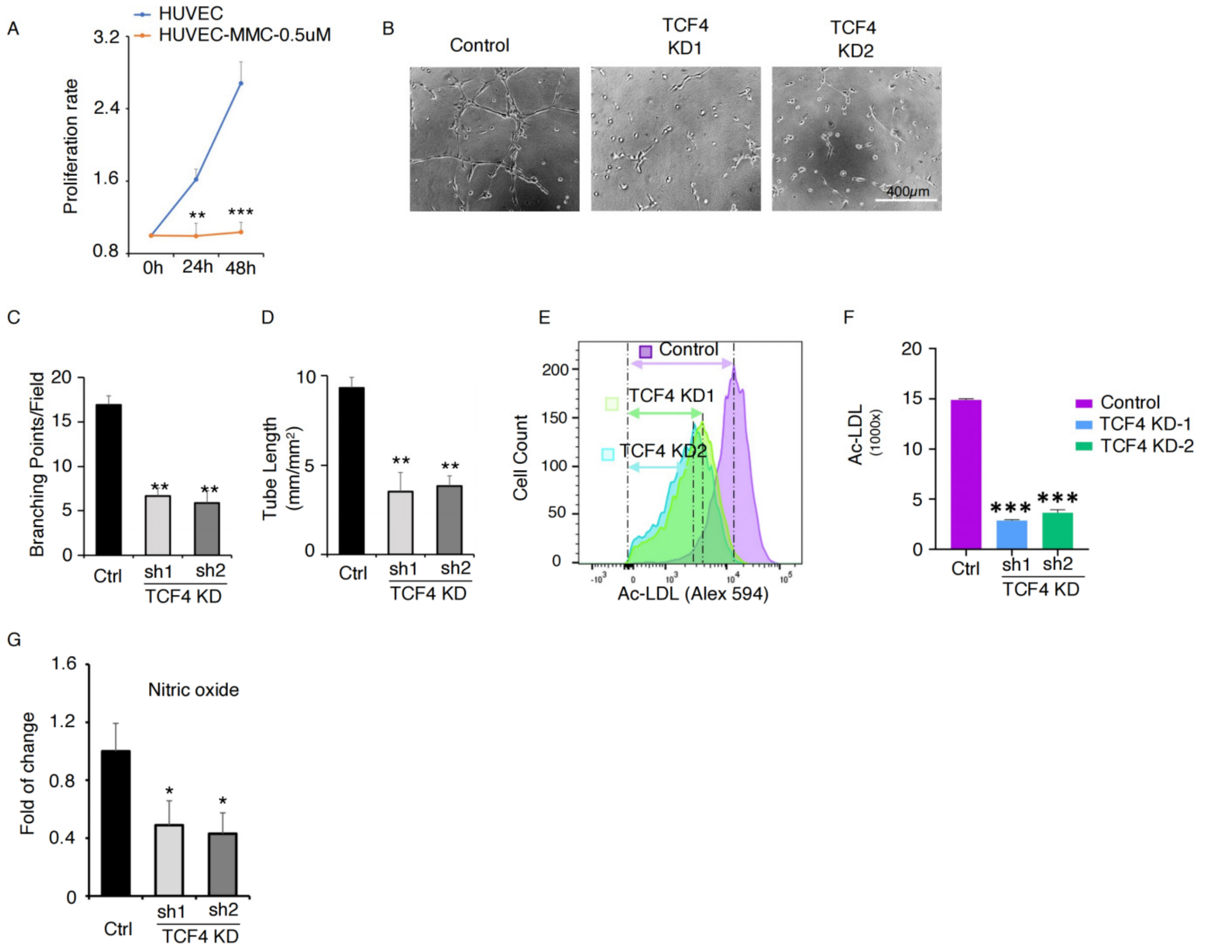


**Figure s3 TCF4 KD significantly impairs EC function without being attributable to impaired cell proliferation**

(**A**) HUVECs proliferation with or without Mitomycin C treatment. (**B**) Representative images of *in vitro* tube formation, scale bars 400 µm. (**C**) The number of EC tube branching points per field. (**D**) The length of EC branching tube. (**E**) Flow cytometry analysis of Ac-LDL uptake. (**F**) Quantification of Ac-LDL uptake. (**G**) Nitrite oxide production by HUVECs. Error bars represent variation between replicates. Data are presented as mean values +/- SD. $n \geq 3$ biologically independent samples. *P<0.05, **P<0.01, ***P<0.001, ****P<0.0001. P values determined by two-tailed Student's t-test.

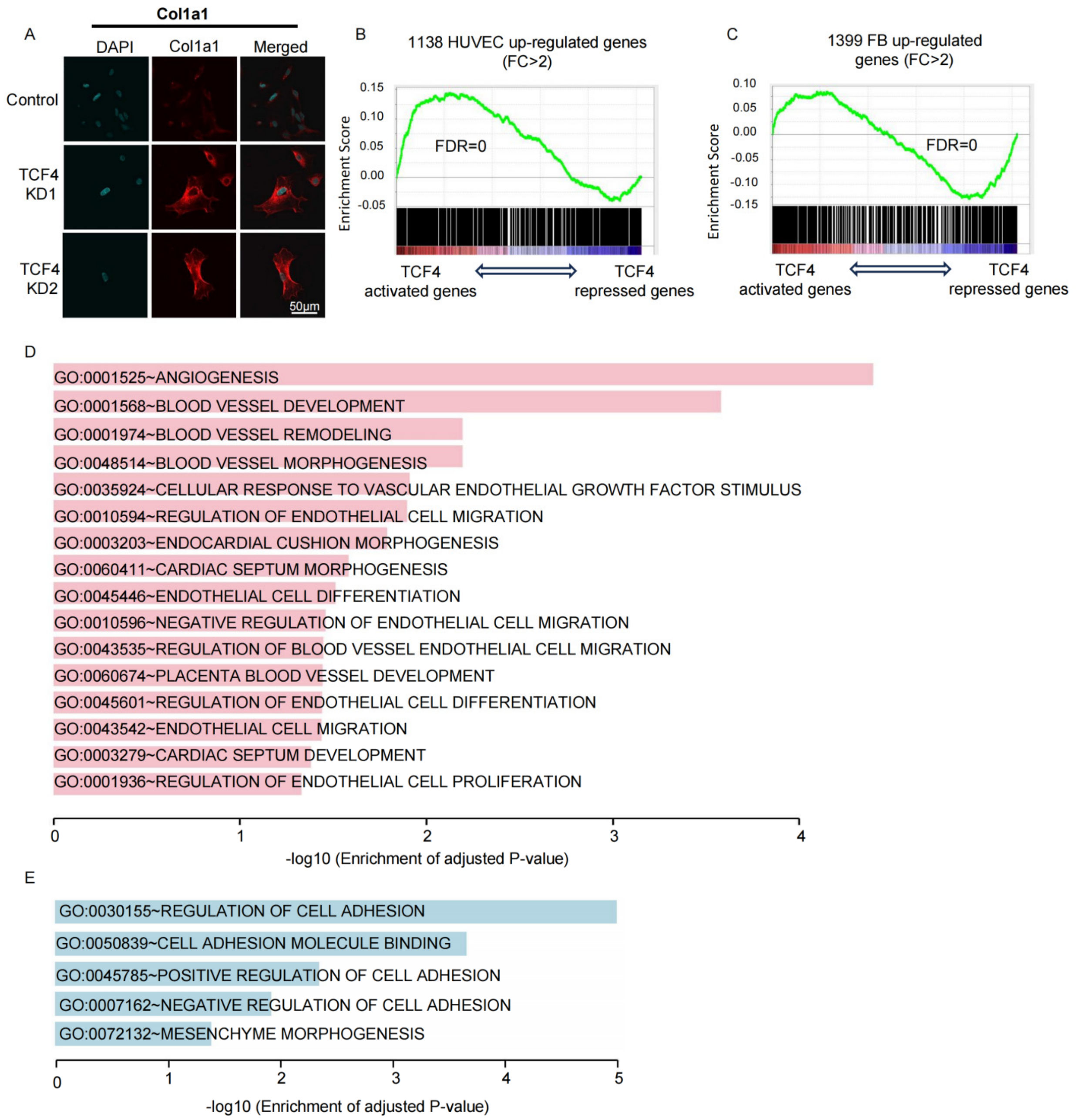


**Figure s4 TCF4 depletion induces EndoMT**

(**A**) Representative immunofluorescence staining images of Col1a1 in control and TCF4 KD HUVECs, scale bars 50 μm. (**B-C**) Gene set enrichment analysis (GSEA) of gene signature on differential expression data from TCF4 KD versus control RNA-seq. On the x-axis, genes are ranked from the most down-regulated (TCF4 activated gene, left end) to the most up-regulated (TCF4 repressed gene, right end) between TCF4 KD and Control samples. HUVEC up-regulated genes (B) are significantly enriched in TCF4-activated genes. Fibroblast up-regulated genes (C) are significantly enriched in TCF4-repressed genes. Error bars represent variation between replicates. (**D-E**) Gene Ontology (GO) enrichment analysis for TCF4 putative target genes based on CUT&RUN in HUVECs.

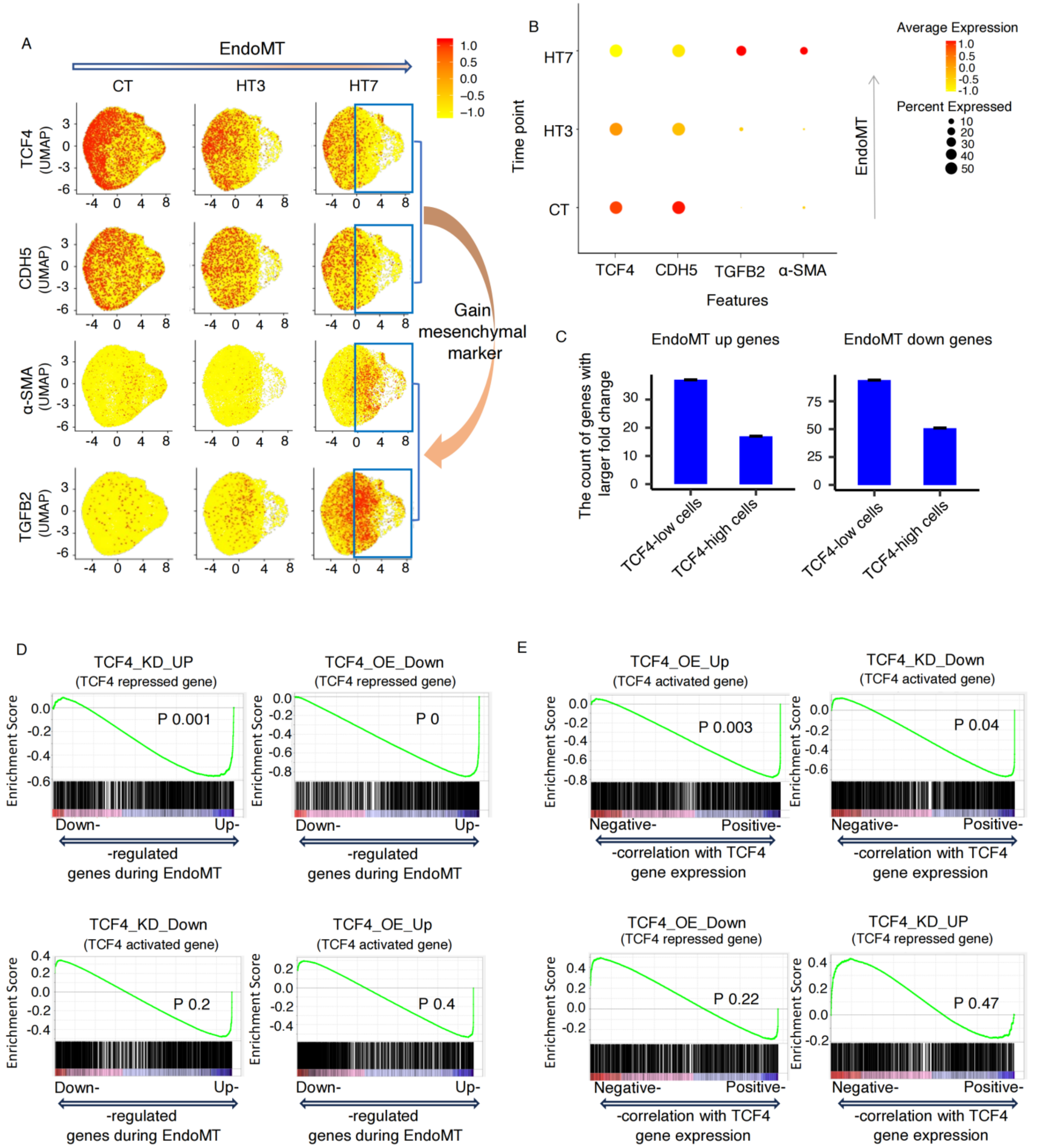


**Figure s5 scRNA-seq of *in vitro* EndoMT suggests high TCF4 expression maintains EC identity during EndoMT**

(**A**) Individual gene expression profiles across cell population at 3 individual time points during EndMT. CT, HT3, and HT7 represent samples collected on day 0, 3, and 7 after treatment with high-glucose and TNFα (HT) (**B**) Average expression and percentage of expressing cells of TCF4, CDH5, TGFβ2, and α-SMA across 3 individual time points during EndoMT. (**C**) Cells with lower TCF4 expression tend to show larger fold changes of gene expression during EndoMT. Cells in the scRNA-seq data were divided to TCF4-low and -high groups based on the

TCF4 expression level. Thereafter, for genes that are differentially expressed between the CT and HT7 samples, we counted the number of genes with larger expression fold change during EndMT in the TCF4-low cells than in the TCF4-high cells, or vice versa. (**D**) GSEA of individual gene sets. On the x-axis, genes are ranked from the most down-regulated (left end) to the most up-regulated genes (right end) between CT and HT7 samples in the scRNA-seq data. The ranked genes were used as the input for GSEA to analyze the enrichment of TCF4-repressed genes (top panel, including TCF4 KD upregulated genes and TCF4 OE downregulated genes) or TCF4-activated genes (bottom panel, including TCF4 KD downregulated genes and TCF4 OE upregulated genes) defined by bulk RNA-seq. (**E**) GSEA of individual gene groups. On the x-axis, genes are ranked based on their expression correlations with TCF4 in the scRNA-seq data. The ranked genes were used as the input for GSEA to analyze the enrichment of TCF4-activated genes (top panel, including TCF4 OE upregulated genes and TCF4 KD downregulated genes) and TCF4-repressed genes (bottom panel, including TCF4 OE downregulated genes and TCF4 KD upregulated genes) defined by bulk RNA-seq. P values were determined by permutation test (D, E).

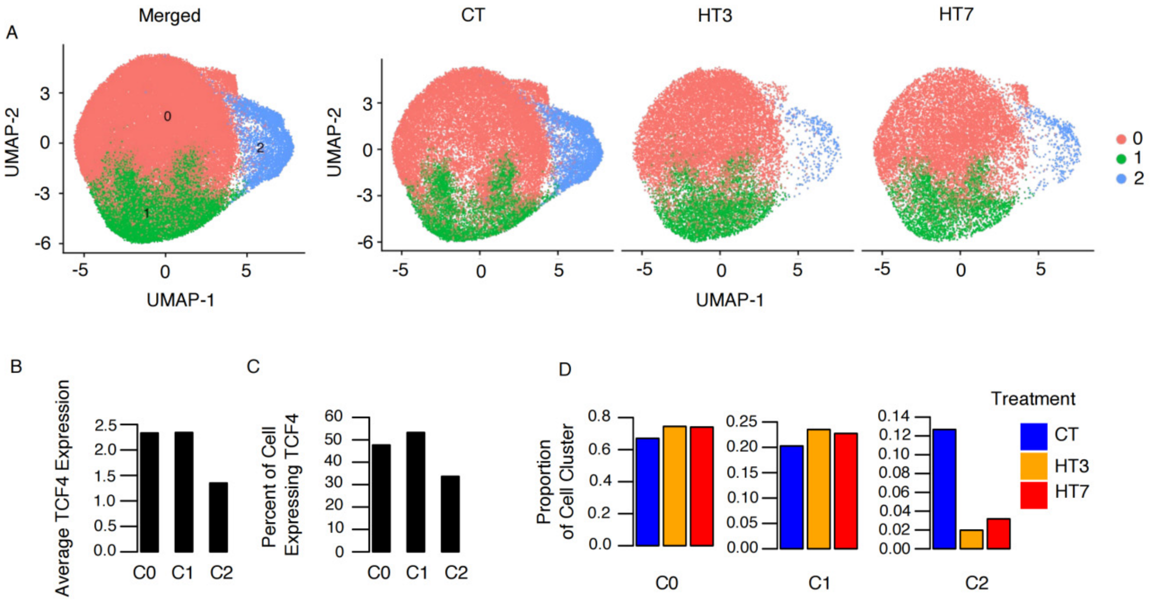


**Figure s6 The proportion of TCF4-low cells decreases as EndoMT progresses**

(A) Cell distribution across individual time points (CT, HT3, and HT7) during EndoMT induction. (**B**) Average TCF4 expression among 3 clusters. (**C**) Percentage of TCF4 expressing cells among 3 clusters. (**D**) Portion of 3 clusters (C0, C1, and C2) at different time points.

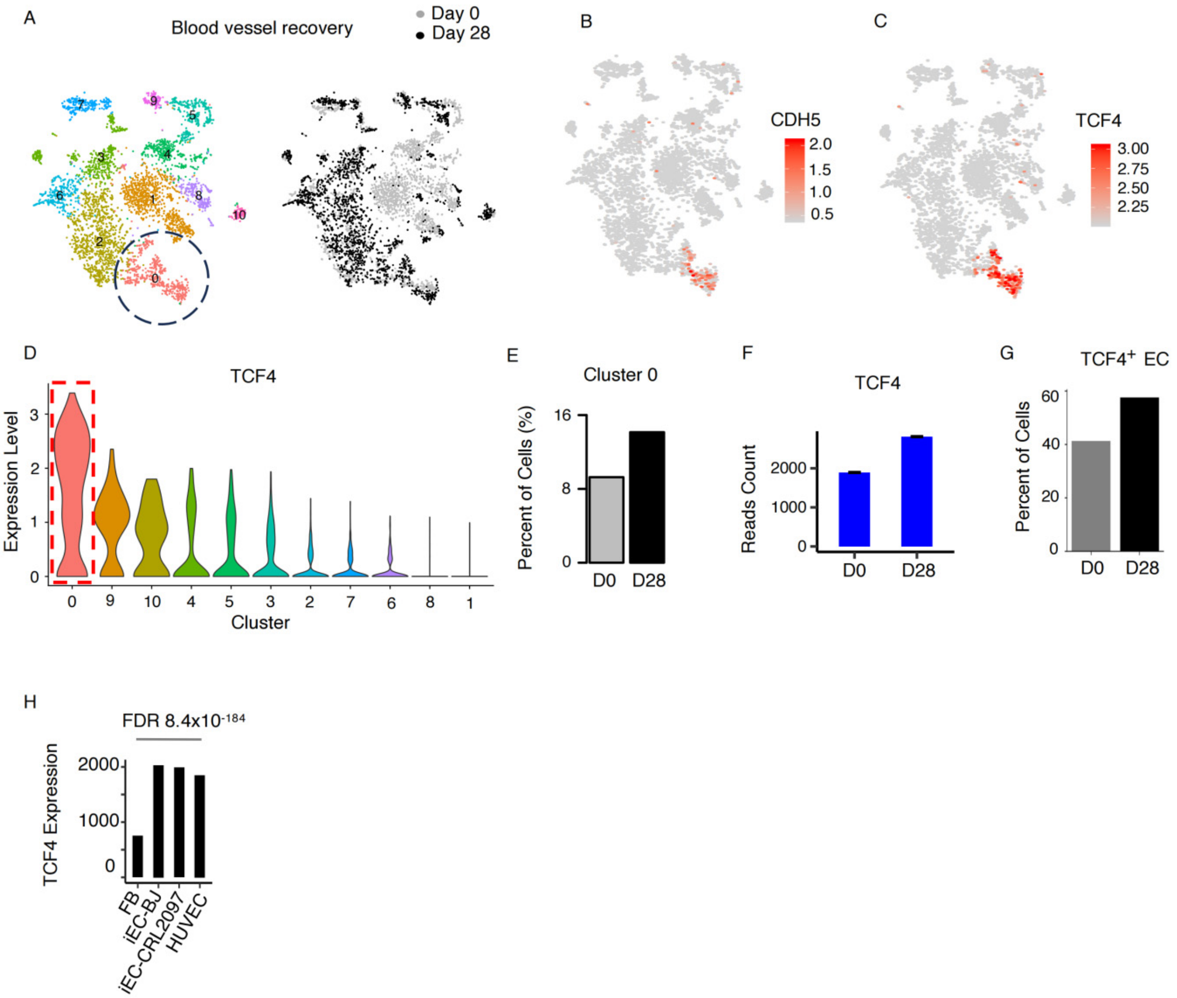


**Figure s7 TCF4 expression is up-regulated in EC transdifferentiated from fibroblast during blood vessel recovery**

(**A**) T-SNE plot of combined cells from days 0 and 28 of *in vivo* of blood vessel recovery model. 11 cell clusters are defined based on transcriptomic similarity. (**B-D**) Expression profiles of CDH5 and TCF4 across 11 individual clusters showed in feature plots (B,C) and VlnPlot (D). (**E**) The proportion of TCF4-positive cell subpopulation (cluster 0) on day 0 and day 28. (**F**) Reads count of TCF4 in cluster 0 on day 0 and day 28. (**G**) Proportion of TCF4 positive EC ($TCF4^+$ EC) among total EC at Day 0 and Day 28. (**H**) Relative expression levels of TCF4 in fibroblast, induced EC from fibroblast (BJ and CRL2097), and HUVECs. N=2 biologically independent samples. P values were determined by two-tailed Negative Binomial test implemented in edgeR v3.14.

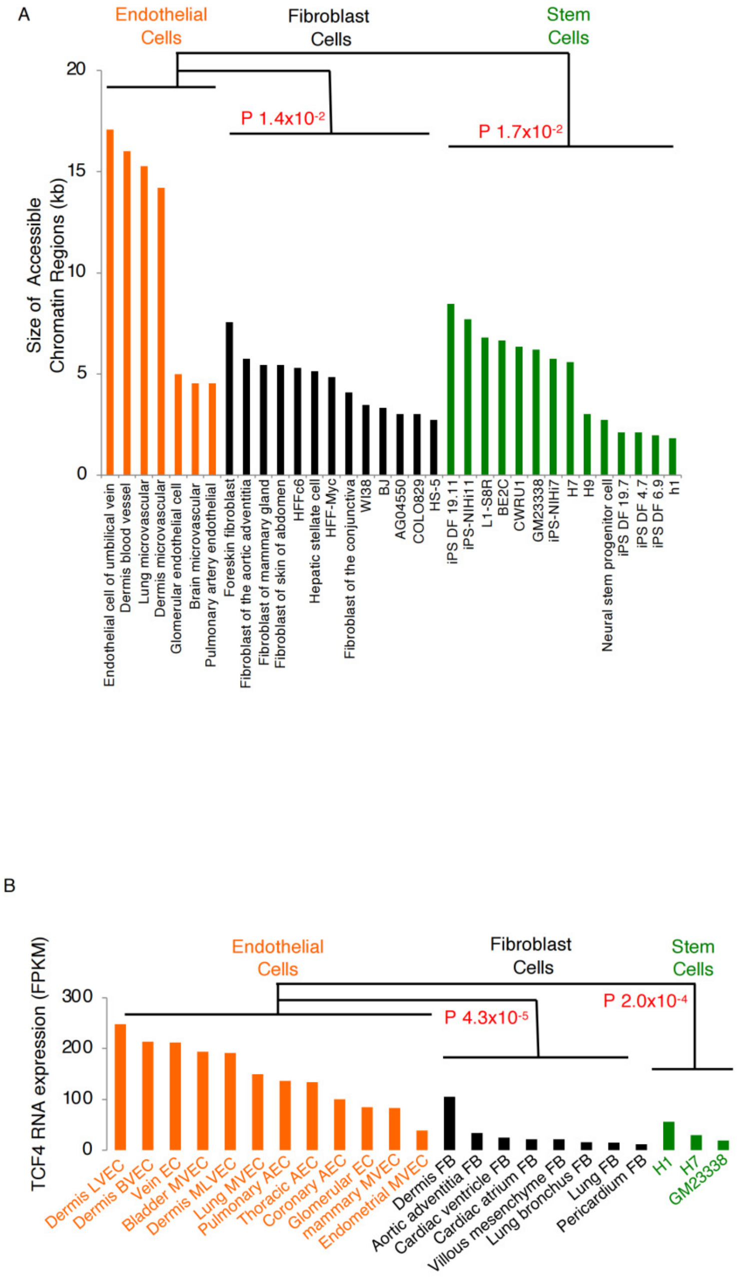


**Figure s8 TCF4 shows stronger chromatin openness and higher gene expression across different EC subtypes compared to fibroblast and stem cells**

(**A**) Summed DNase peak width within +/- 10kb around TCF4 gene body across endothelial cells, fibroblast, and stem cell subtypes. (**B**) TCF4 expression level across subtypes of endothelial cells, fibroblasts, and stem cells. P values were determined by Two-tailed Wilcoxon Rank Sum test.

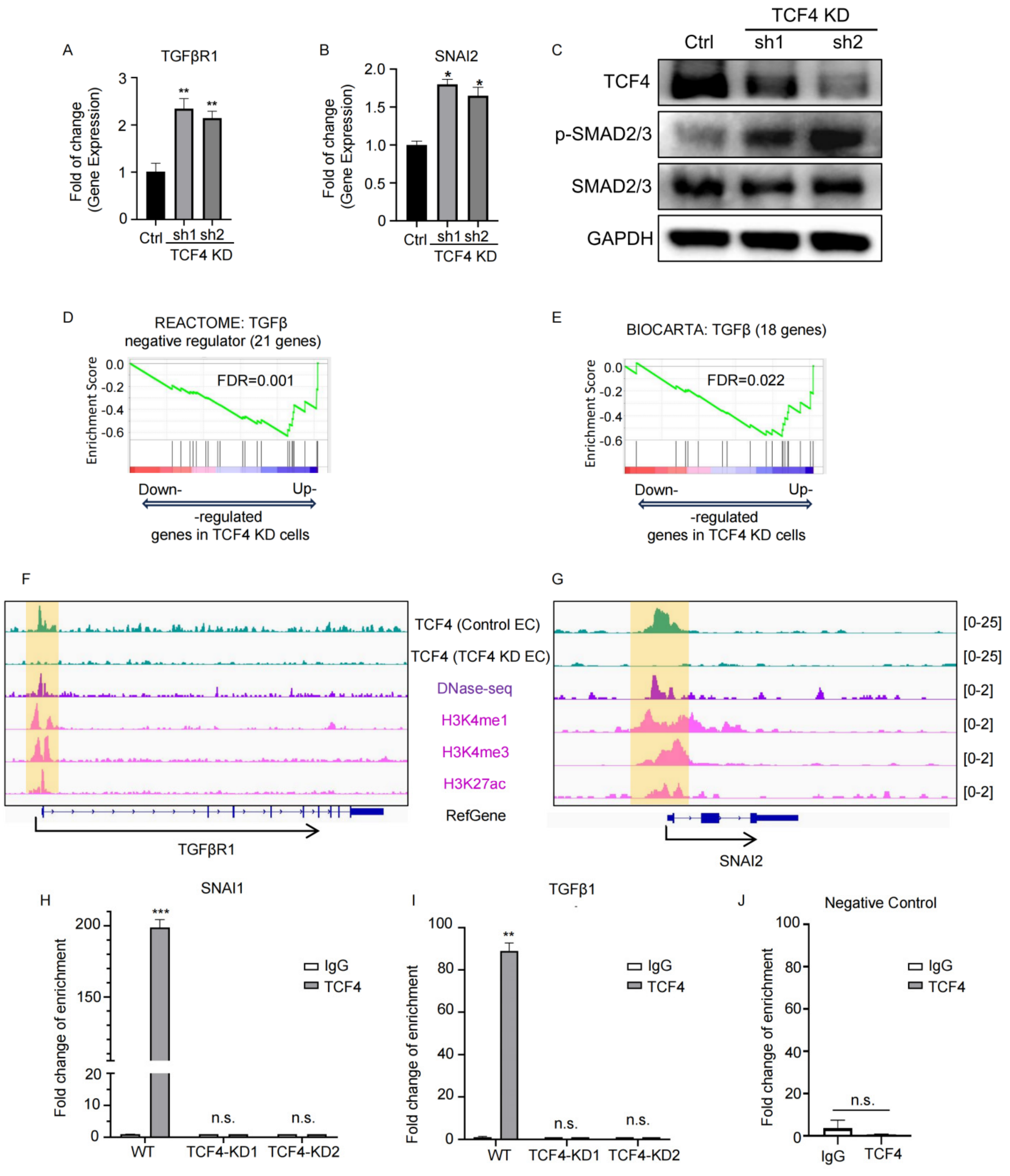


**Figure s9 TCF4 bind promoter to regulate TGFβ signaling pathway**

(**A-B**) qPCR results showing gene expression changes of TGFβR1 and SNAI2 after TCF4 KD in HUVECs. (**C**) Western blot results showing p-SMAD2/3 and SMAD2/3 protein expression after TCF4 KD in HUVECs. (**D-E**) GSEA of gene signature on differential expression data from TCF4 KD versus control RNA-seq. Results for gene sets of TGFβ signaling pathways from 2 different sources are shown. (**F-G**) Genomic tracks of CUT&RUN showing the TCF4 binding at TGFβR1 (F) and SNAI2 gene loci (G), along with tracks of DNase-seq and ChIP-seq of related

histone modifications. (**H-J**) TCF4 ChIP-qPCR with IgG as the control in both Control and TCF4 KD HUVECs for TCF4-to-SNAI1 binding site (H), TCF4-to-TGFβ1 binding site (I), and negative control sites (J). Error bars represent variation between replicates.

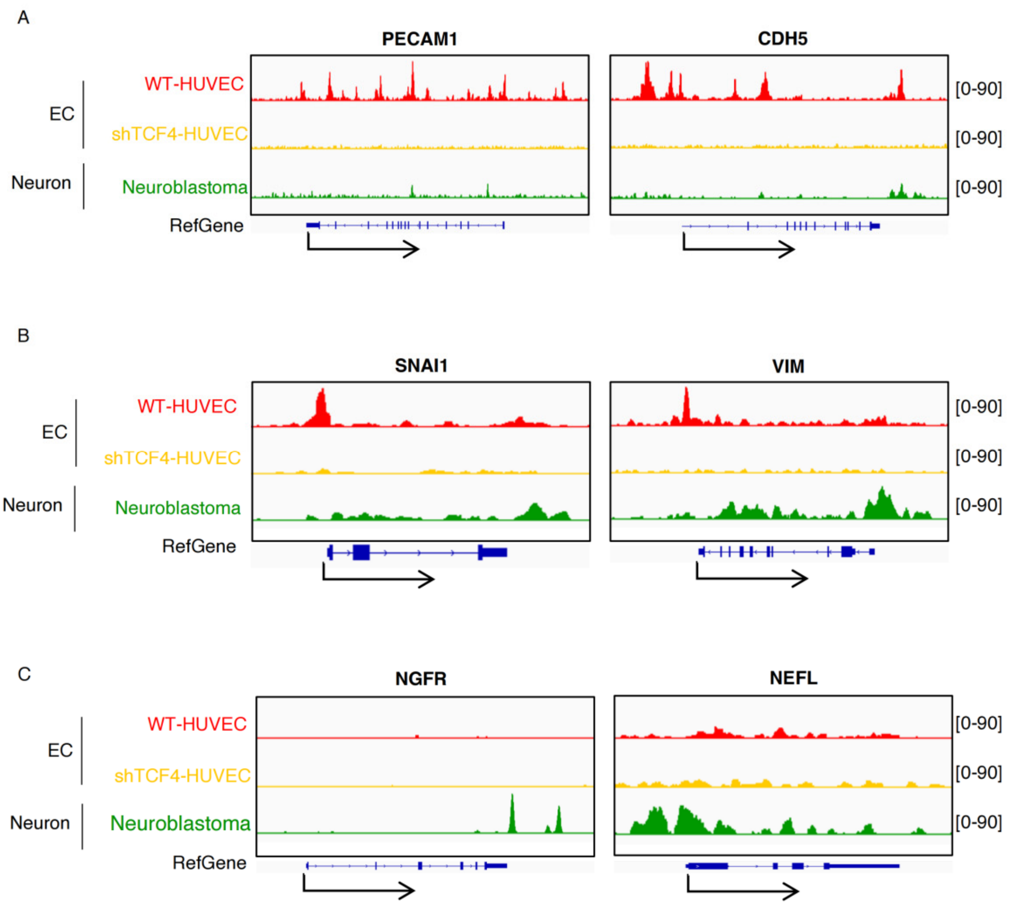


**Figure s10 TCF4 displays cell type-specific binding in endothelial and neuronal cells**

CUT&RUN analysis of TCF4 reveals binding peaks at (**A**) endothelial cell markers (PECAM1 and CDH5), (**B**) mesenchymal markers (SNAI1 and VIM), and (**C**) neuronal markers (NGFR and NEFL) in different cell types.

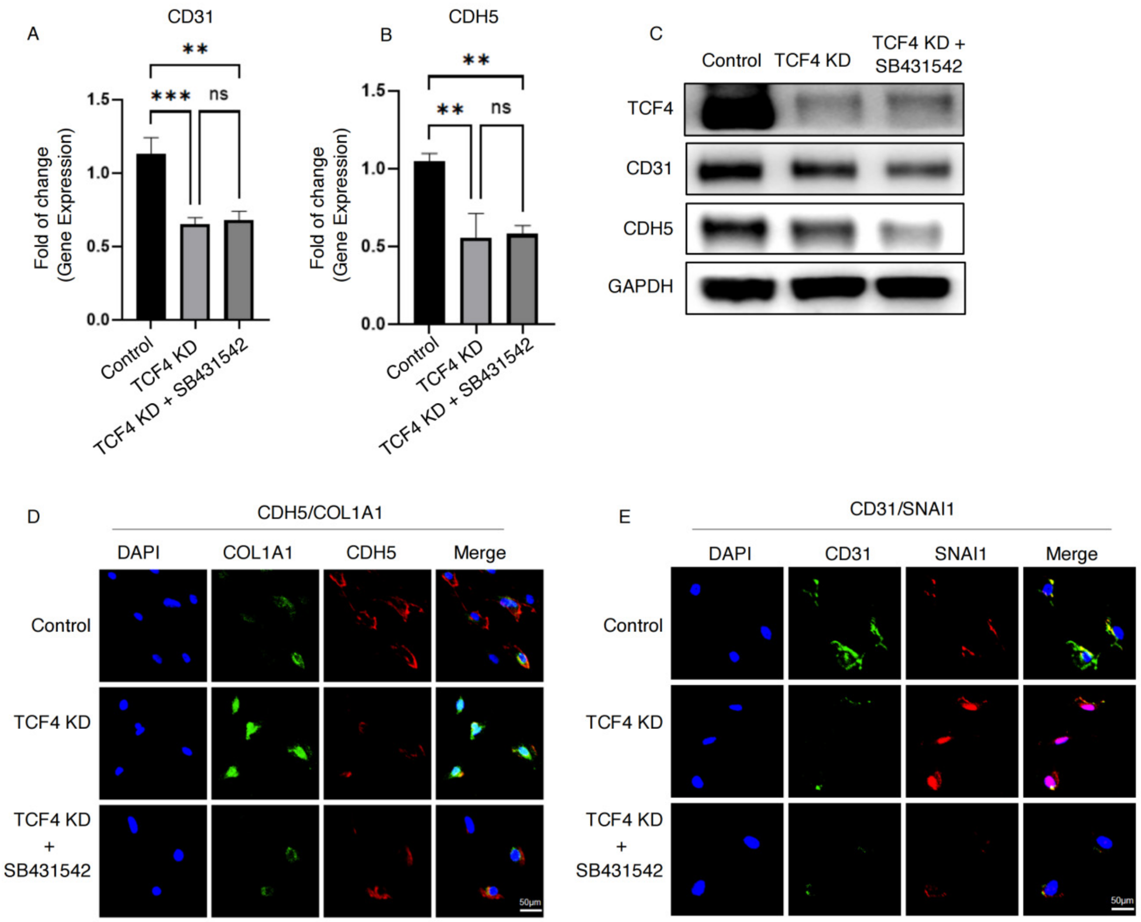


**Figure s11 TGFβR inhibitor reverses the mesenchymal phenotype induced by TCF4 KD, but does not restore EC marker expression**

(**A-E**) qPCR results (A-B) showing gene expression level changes, western blot (C) and immunofluorescence staining (D-E) showing protein expression level change, after TGFβR inhibitor (SB431542) treatment with or without TCF4 KD, scale bars 50 μm.

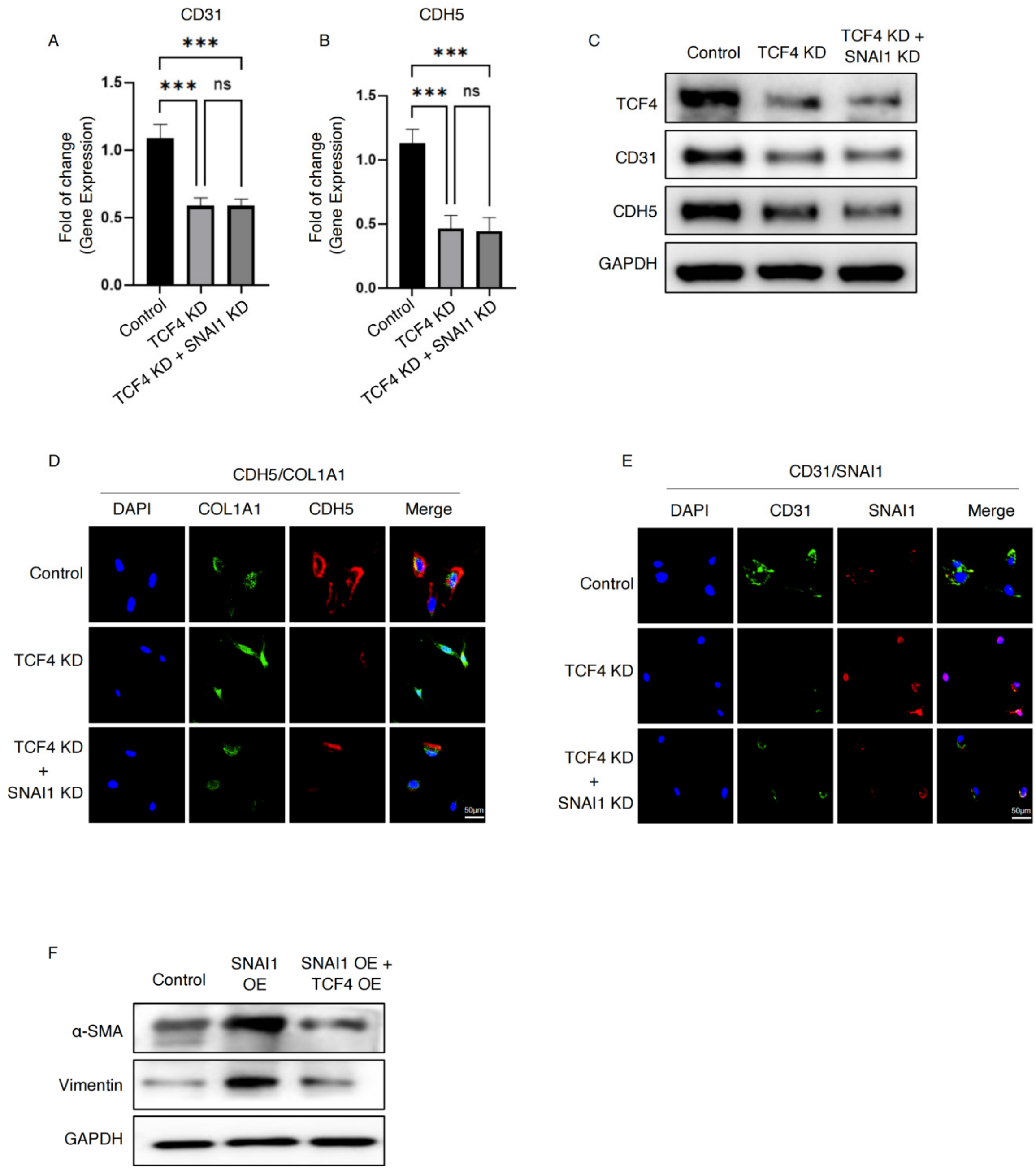


**Figure s12 SNAI1 KD reverses the mesenchymal phenotype induced by TCF4 KD, but does not restore EC marker expression**

(**A-E**) qPCR results (A-B) showing gene expression level changes, western blot (C) and immunofluorescence staining (D-E) showing protein expression level change, after TCF4 KD with or without SNAI1 KD, scale bars 50 μm. (**F**)Western blot analysis showing changes in protein expression levels following SNAI1 overexpression (OE), with or without co-overexpression of TCF4.

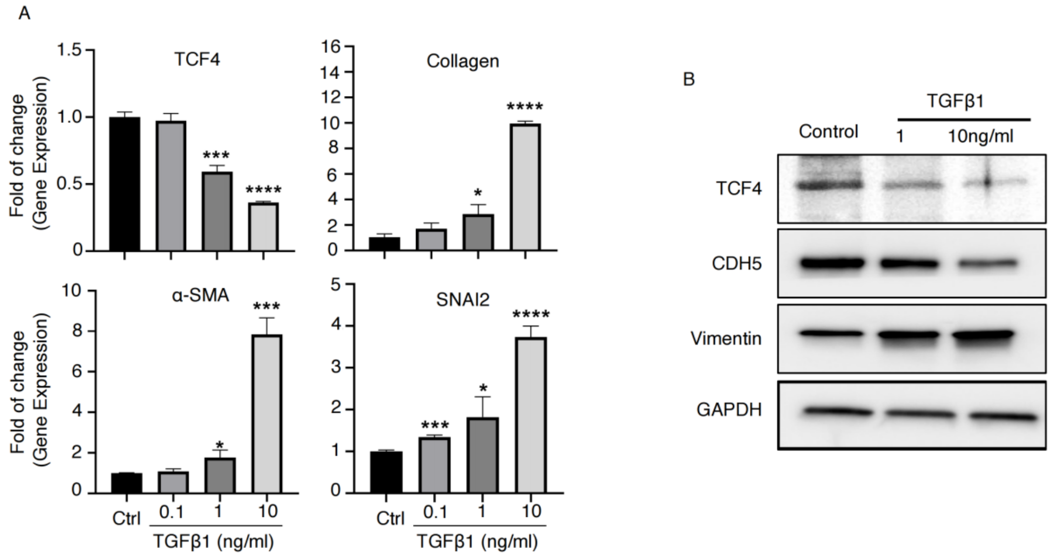


**Figure s13 TCF4 is down-regulated during TGFβ-induced EndoMT**

(**A-B**) qPCR results showing gene expression changes (A), Western blot results showing protein level changes (B) in HUVECs treated with different doses of TGFβ1. Error bars represent variation between replicates. Data are presented as mean values +/- SD. n≥3 biologically independent samples. *P<0.05, **P<0.01, ***P<0.001, ****P<0.0001. P values determined by two-tailed Student's t-test.

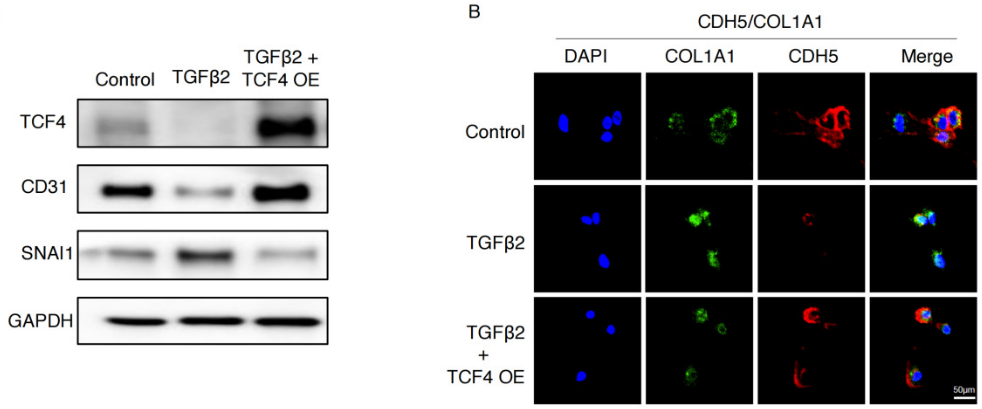


**Figure s14 TCF4 overexpression alleviates TGFβ2 induced EndoMT**

(**A-B**) Western blot (A) and immunofluorescence staining (B) showing protein expression level change, after TGFβ2 treatment with or without TCF4 OE, scale bars 50 μm.

Supplementary table1: Sequences of shTCF4

| Name | Sequences (5'-->3') |
|---|---|
| TCF4-sh1 | GAAAGGAATCTGAATCCGAAA |
| TCF4-sh2 | GAGACTGAACGGCAATCTTTC |

Supplementary table2: qPCR primer sequences

| Name | Sequences (5'-->3') |
|---|---|
| GAPDH-F | GTCTCCTCTGACTTCAACAGCG |
| GAPDH-R | ACCACCCTGTTGCTGTAGCCAA |
| TCF4-2F | CCTGGCTATGCAGGAATGTT |
| TCF4-2R | TCTGCTGAGGAGTGTGATGG |
| TCF4-3F | TGCCATGGAGGTACAGACAA |
| TCF4-3R | ATAGCCTGGCGAGTCCCTAT |
| CD31-F | AACAGTGTTGACATGAAGAGCC |
| CD31-R | TGTAAAACAGCACGTCATCCTT |
| CDH5-F | TTGGAACCAGATGCACATTGAT |
| CDH5-R | TCTTGCGACTCACGCTTGAC |
| CD34-F | CTACAACACCTAGTACCCTTGGA |
| CD34-R | GGTGAACACTGTGCTGATTACA |
| αSMA-F | CTATGCCTCTGGACGCACAACT |
| αSMA-R | CAGATCCAGACGCATGATGGCA |
| COL1A1-F | GATTCCCTGGACCTAAAGGTGC |
| COL1A1-R | AGCCTCTCCATCTTTGCCAGCA |
| N-Cadherin-F | CCTCCAGAGTTTACTGCCATGAC |
| N-Cadherin-R | GTAGGATCTCCGCCACTGATTC |
| Vimentin-F | AGGCAAAGCAGGAGTCCACTGA |
| Vimentin-R | ATCTGGCGTTCCAGGGACTCAT |
| TGFβ1-F | GGCCAGATCCTGTCCAAGC |
| TGFβ1-R | GTGGGTTTCCACCATTAGCAC |
| TGFβR-F | ACGGCGTTACAGTGTTTCTG |
| TGFβR-R | GCACATACAAACGGCCTATCTC |
| SNAI1-F | TGCCCTCAAGATGCACATCCGA |
| SNAI1-R | GGGACAGGAGAAGGGCTTCTC |
| TGFβ-ChIP-F | GCCCCACTGTAGATGGTGTC |
| TGFβ-ChIP-R | GGATTTTGCCATGTGCCCAG |
| SNAI-ChIP-F | AGTGGTTCTTCTGCGCTACT |
| SNAI-ChIP-R | AGTTAGGCTTCCGATTGGGG |
| CDH5-ChIP-1-F | CTTCCGCTGGACAAGGTTTC |
| CDH5-ChIP-1-R | AGGTGCAGTCAGGTTGAGGC |
| CDH5-ChIP-2-F | TTGTGCCCTCCTGTTACCTCC |
| CDH5-ChIP-2-R | CACTGGCCTGTGCATTGCTT |
| CDH5-ChIP-3-F | TTTCCTGTTGTTCCCATTAC |
| CDH5-ChIP-3-R | GATTCTTTCTTCCTGCCTCA |
| CDH5-ChIP-4-F | AATTGTGCCTTGAATCAGCC |

| | |
|---|---|
| CDH5-ChIP-4-R | CTTGGAGCCCTGTCCTTGTC |
| CDH5-ChIP-5-F | AAAGCATAGGGAAGAGTGAAA |
| CDH5-ChIP-5-R | CCTGCATAGCTCCAGAAAGC |

Supplementary table3: resources

| **Antibodies** | **Source** | **Identifier** |
|---|---|---|
| VE-cadherin | Cell Signaling TECHNOLOGY | 2500S |
| α-SMA | Cell Signaling TECHNOLOGY | 19245S |
| COL1A | Santa Cruz | sc-59772 |
| PECAM1 | Cell Signaling TECHNOLOGY | 3528S |
| Vimentin | Cell Signaling TECHNOLOGY | 5741S |
| Goat anti-Rabbit IgG (H+L) Alexa Fluor™ Plus 594 | Invitrogen | A32740 |
| Goat anti-Mouse IgG (H+L) Alexa Fluor™ Plus 488 | Invitrogen | A32723 |
| TCF4 | Abcam | Ab217668 |
| GAPDH | Santa Cruz Biotechnology | sc-32233 |
| Goat anti-Rabbit IgG | GenDEPOT | SA002-500 |
| Goat anti-Mouse IgG | GenDEPOT | SA001-500 |
| HRP Substrate | Millipore | WBKLS0100 |
